# Supplementary material for: Towards resilience: Transcriptional insights on flavonoid biosynthesis during peanut seed maturation phases
Source: PLoS One. 2025 Jul 1;20(7):e0325686. doi: 10.1371/journal.pone.0325686 (PMC12212506; doi:10.1371/journal.pone.0325686)
Supplement: S1 File — S1 Table. Quality control of RNA samples extracted from fresh peanut seeds. S2 Table. Reads mapped to the peanut genome. S3 Table. Primers designed for RT-qPCR study. S1 File. Significant genes associated with the RNA-seq. (ZIP) [file pone.0325686.s001.zip › S1 Table RNA Quality Control.docx]

**Table S1.** Quality control of RNA samples extracted from fresh peanut seeds.

| Sample ID | Biological replicate | Total RNA | | NanoDrop^®^ absorbances | | RIN ^6^ |
| --- | --- | --- | --- | --- | --- | --- |
|  |  | ng/µL ^2^ | µg/µL ^3^ | 260/280 ^4^ | 260/230 ^5^ |  |
| Pool of seed stages ^1^ | 1 | 49.4 | 1.9 | 2.12 | 1.93 | 8.4 |
|  | 2 | 107.2 | 1.2 | 2.13 | 2.12 | 7.4 |
|  | 3 | 60.7 | 1.1 | 2.10 | 2.03 | 7.0 |
| R5 | 1 | 212.3 | 1.4 | 2.13 | 2.23 | 8.1 |
|  | 2 | 267.2 | 3.0 | 2.15 | 2.09 | 7.8 |
|  | 3 | 267.1 | 1.7 | 2.13 | 2.25 | 7.7 |
| R6 | 1 | 219.8 | 5.9 | 2.14 | 2.27 | 9.2 |
|  | 2 | 254.3 | 7.5 | 2.15 | 2.11 | 9.1 |
|  | 3 | 308.1 | 7.5 | 2.16 | 2.30 | 9.3 |
| R7 | 1 | 100.7 | 6.2 | 2.12 | 2.15 | 7.8 |
|  | 2 | 140.8 | 7.1 | 2.11 | 2.17 | 7.3 |
|  | 3 | 77.6 | 8.6 | 2.14 | 2.03 | 8.5 |
| R8 | 1 | 87.5 | 2.8 | 2.14 | 2.14 | 8.5 |
|  | 2 | 72.9 | 3.9 | 2.11 | 2.06 | 8.5 |
|  | 3 | 150.2 | 2.2 | 2.15 | 2.21 | 7.9 |
| R9 | 1 | 67.0 | 2.5 | 2.11 | 2.02 | 7.6 |
|  | 2 | 43.6 | 2.0 | 2.17 | 1.84 | 7.9 |
|  | 3 | 39.8 | 4.2 | 2.14 | 1.86 | 8.8 |

^1^ Pool of samples from all seed stages (R5, R6, R7, R8, and R9)

^2^ Total RNA with concentration in micrograms per microliter (µg/µL) in a volume of 28 microliters (discounting 2 µL used to measure absorbance in the NanoDrop^®^).

^3^ The recommendation is that the total RNA concentration be at least 1.0 µg/µL.

^4,5^ Absorbances obtained with values ​​above 1.8 indicating high quality of the extracted RNA.

^6^ RNA integrity obtained using the RNAScreenTape equipment. The recommendation is that RIN values (RNA Integrity Number) ​​be greater than or equal to 7.0.
