# Supplementary material for: Towards resilience: Transcriptional insights on flavonoid biosynthesis during peanut seed maturation phases
Source: PLoS One. 2025 Jul 1;20(7):e0325686. doi: 10.1371/journal.pone.0325686 (PMC12212506; doi:10.1371/journal.pone.0325686)
Supplement: S1 File — S1 Table. Quality control of RNA samples extracted from fresh peanut seeds. S2 Table. Reads mapped to the peanut genome. S3 Table. Primers designed for RT-qPCR study. S1 File. Significant genes associated with the RNA-seq. (ZIP) [file pone.0325686.s001.zip › S2 Table Mapping Reads.docx]

**Table S2.** Mapping reads to the peanut genome.

| Seed stages | Biological replicate ^1^ | Mapped reads ^2^ | Total reads ^3^ | % Mapped reads ^4, 5^ |
| --- | --- | --- | --- | --- |
| R5 | 1 | 17761951 | 33818614 | 52.52 |
|  | 2 | 11966822 | 22618294 | 52.91 |
|  | 3 | 12495931 | 23548846 | 53.06 |
| R6 | 1 | 10714489 | 20455495 | 52.38 |
|  | 2 | 19853901 | 36722045 | 54.07 |
|  | 3 | 23754413 | 44921732 | 52.88 |
| R7 | 1 | 10464928 | 16266747 | 64.33 |
|  | 2 | 14816145 | 23401228 | 63.31 |
|  | 3 | 12161636 | 19247983 | 63.18 |
| R8 | 1 | 16779044 | 25324144 | 66.26 |
|  | 2 | 31035771 | 42706969 | 72.67 |
|  | 3 | 15709163 | 24274858 | 64.71 |
| R9 | 1 | 15000808 | 22362789 | 67.08 |
|  | 2 | 13876681 | 20531293 | 67.59 |
|  | 3 | 27894528 | 39114260 | 71.32 |

^1^ Fresh seed sample

^2^ Uniquely mapped reads number

^3^ Number of input reads

^4^ Uniquely mapped reads (Mapped Reads / Total reads)

^5^ Annotation: arahy.Tifrunner.gnm1.ann1.CCJH.gene_models_main.gff3
