# Supplementary material for: Towards resilience: Transcriptional insights on flavonoid biosynthesis during peanut seed maturation phases
Source: PLoS One. 2025 Jul 1;20(7):e0325686. doi: 10.1371/journal.pone.0325686 (PMC12212506; doi:10.1371/journal.pone.0325686)
Supplement: S1 File — S1 Table. Quality control of RNA samples extracted from fresh peanut seeds. S2 Table. Reads mapped to the peanut genome. S3 Table. Primers designed for RT-qPCR study. S1 File. Significant genes associated with the RNA-seq. (ZIP) [file pone.0325686.s001.zip › S3 Table Primers.docx]

**Table S3.** Primers designed for RT-qPCR study.

| Alias ^1^ | Gene ID | Left Primer (Forward) ^2^ | Right Primer (Reverse) ^2^ |
| --- | --- | --- | --- |
| *AhCHS* | arahy.Tifrunner.gnm1.ann1.0FI6RG | ACCTGAGAAGATGAGAGCCA | ATTCGAGTCCTTCACCAGTG |
| *AhCHI* | arahy.Tifrunner.gnm1.ann1.VJQ7J1 | ATCGTTACAGGTCCGTTTGA | CATTGGCTTCTTGGTCAGTG |
| *AhFLS* | arahy.Tifrunner.gnm1.ann1.4Y1607 | AGCAACCAGGAATCACAACA | TTGGGATCTCATGGTTGACG |
| *AhLDOX* | arahy.Tifrunner.gnm1.ann1.AQ6B1J | AAGTTCCGACCATTGACCTC | CATGGTTGACAAGGTGCATC |
| *AhANR* | arahy.Tifrunner.gnm1.ann1.IK60LM | ATGTGGAGGATATTTGCCGG | GCAAGCTCAGGAACACTAGT |
| *AhMYB12* | arahy.Tifrunner.gnm1.ann1.PR7AYB | TACCAGGCAGAACAGACAAC | AGATCTTGGGAATGGCAGTG |
| *AhMYB308* | arahy.Tifrunner.gnm1.ann1.TG6F30 | TAGGGTTGCATAGAGGTCCA | CCACATCTCAAAAGTCCAGC |
| *Ah60S* | arahy.Tifrunner.gnm1.ann1.DS63LJ | CGAGGTGTTACAGGCAAATC | CCGCACCCTTCTTCTTCATT |

^1^ *Arachis hypogaea (Ah)*

^2^ The primers were designed using Primer 3 software (<https://primer3.ut.ee/>).
