# Supplementary material for: Towards resilience: Transcriptional insights on flavonoid biosynthesis during peanut seed maturation phases
Source: PLoS One. 2025 Jul 1;20(7):e0325686. doi: 10.1371/journal.pone.0325686 (PMC12212506; doi:10.1371/journal.pone.0325686)
Supplement: Attachment — Submitted filename: PONE-D-25-12406_reviewer.pdf [file pone.0325686.s002.pdf]

PLOS ONE

Towards Resilience: Transcriptional Insights on Flavonoid Biosynthesis During Peanut Seed Maturation Phases

--Manuscript Draft--

**Manuscript Number:**	PONE-D-25-12406
**Article Type:**	Research Article
**Full Title:**	Towards Resilience: Transcriptional Insights on Flavonoid Biosynthesis During Peanut Seed Maturation Phases
**Short Title:**	Flavonoid Biosynthesis During Peanut Seed Maturation
**Corresponding Author:**	Gustavo Fonseca de Oliveira<br>Sao Paulo State University Julio de Mesquita Filho: Universidade Estadual Paulista Julio de Mesquita Filho<br>Botucatu, BRAZIL
**Keywords:**	Arachis hypogaea L.; anthocyanin; MYB; chalcone; seed development
**Abstract:**	Flavonoids are secondary metabolites widely studied as a metabolic protector against several stressors in plants, yet they are understudied in the events of peanut seed development. Substantial advances in the understanding of peanut seed maturation have been made in recent years, however, the role of flavonoids in this process is unclear. Here, the fundamental question asked was: are flavonoids involved in peanut seed maturation phases? This study investigates whether the main transcripts associated with the flavonoid pathway, such as anthocyanins, are biologically linked to the physiological quality components of peanut seeds during development. For this purpose, peanut seeds classified into five stages (R5, R6, R7, R8 and R9) which were used for the evaluation of quality attributes, such as desiccation tolerance, vigor and longevity parameters, and for RNA sequencing (RNAseq). Interestingly, anthocyanins accumulated more in the beginning of the seed filling phase, coinciding with the upregulation in RNAseq and quantitative PCR of key genes in its pathway, such as AhCHS (0FI6RG), AhCHI (VJQ7J1), AhFLS (4Y1607), AhLDOX (AQ6B1J) and AhANR (IK60LM). Additionally, we found that AhMYB12 (PR7AYB) and AhMYB308 (TG6F30) exhibited increased expression at the early stages (R5 and R6) and decreased at the later ones (R7, R8 and R9), respectively. AhCHS expression acts in synergy with well-known seed maturation regulators, such as the ABA response (e.g., ABI5 and ABI3). The involvement of flavonoid biosynthesis in peanut seed development is suggested here as a contributor to its resilience during the acquisition of physiological quality attributes, highlighting the molecular aspects associated with survival in the dry state.
**Order of Authors:**	Gustavo Roberto Fonseca de Oliveira
	Liam Walker, Researcher Fellow
	Rômulo Pedro Macêdo Lima, Researcher
	Thiago Barbosa Batista, Researcher
	Clissia Barboza Mastrangelo, Researcher
	Edvaldo Aparecido Amaral da Silva, Professor
**Additional Information:**	
**Question**	**Response**
**Financial Disclosure**<br><br>Enter a financial disclosure statement that describes the sources of funding for the work included in this submission. Review the submission guidelines for detailed requirements. View published research	Yes

Powered by Editorial Manager® and ProduXion Manager® from Aries Systems Corporation

articles from *PLOS ONE* for specific examples.<br><br>This statement is required for submission and **will appear in the published article** if the submission is accepted. Please make sure it is accurate.<br><br>**Funded studies**<br>Enter a statement with the following details:<br>• Initials of the authors who received each award<br>• Grant numbers awarded to each author<br>• The full name of each funder<br>• URL of each funder website<br>• Did the sponsors or funders play any role in the study design, data collection and analysis, decision to publish, or preparation of the manuscript?<br><br>Did you receive funding for this work?	
Please add funding details.<br>as follow-up to "**Financial Disclosure**<br><br>Enter a financial disclosure statement that describes the sources of funding for the work included in this submission. Review the submission guidelines for detailed requirements. View published research articles from *PLOS ONE* for specific examples.<br><br>This statement is required for submission and **will appear in the published article** if the submission is accepted. Please make sure it is accurate.	This study was funded by São Paulo Research Foundation – FAPESP grant number #2020/14050-3 and #2023/00435-9 for G.R. Fonseca de Oliveira, #2021/07331 for E.A. Amaral da Silva.

**Funded studies**<br>Enter a statement with the following details:<br>• Initials of the authors who received each award<br>• Grant numbers awarded to each author<br>• The full name of each funder<br>• URL of each funder website<br>• Did the sponsors or funders play any role in the study design, data collection and analysis, decision to publish, or preparation of the manuscript?<br><br>Did you receive funding for this work?"	
Please select the country of your main research funder (please select carefully as in some cases this is used in fee calculation).<br>as follow-up to "**Financial Disclosure**<br><br>Enter a financial disclosure statement that describes the sources of funding for the work included in this submission. Review the submission guidelines for detailed requirements. View published research articles from *PLOS ONE* for specific examples.<br><br>This statement is required for submission and **will appear in the published article** if the submission is accepted. Please make sure it is accurate.<br><br>**Funded studies**<br>Enter a statement with the following details:<br>• Initials of the authors who received each award<br>• Grant numbers awarded to each author<br>• The full name of each funder<br>• URL of each funder website<br>• Did the sponsors or funders play any role in the study design, data collection and analysis, decision to publish, or preparation of the manuscript?<br><br>Did you receive funding for this work?"	BRAZIL - BR
**Competing Interests**	The authors have declared that no competing interests exist.

Use the instructions below to enter a competing interest statement for this submission. On behalf of all authors, disclose any competing interests that could be perceived to bias this work—acknowledging all financial support and any other relevant financial or non-financial competing interests.<br><br>This statement is required for submission and **will appear in the published article** if the submission is accepted. Please make sure it is accurate and that any funding sources listed in your Funding Information later in the submission form are also declared in your Financial Disclosure statement.<br><br>View published research articles from *PLOS ONE* for specific examples.<br><br>**NO authors have competing interests**<br><br>Enter: *The authors have declared that no competing interests exist.*<br><br>**Authors with competing interests**<br><br>Enter competing interest details beginning with this statement:<br><br>*I have read the journal's policy and the authors of this manuscript have the following competing interests: [insert competing interests here]*<br><br>* typeset	
**Ethics Statement**<br><br>Enter an ethics statement for this submission. This statement is required if the study involved:<br><br>• Human participants<br>• Human specimens or tissue<br>• Vertebrate animals or cephalopods<br>• Vertebrate embryos or tissues	N/A

Powered by Editorial Manager® and ProduXion Manager® from Aries Systems Corporation

• Field research<br><br>Write "N/A" if the submission does not require an ethics statement.<br><br>General guidance is provided below. Consult the submission guidelines for detailed instructions. **Make sure that all information entered here is included in the Methods section of the manuscript.**	

Powered by Editorial Manager® and ProduXion Manager® from Aries Systems Corporation

**Format for specific study types**<br><br>**Human Subject Research (involving human participants and/or tissue)**<br>• Give the name of the institutional review board or ethics committee that approved the study<br>• Include the approval number and/or a statement indicating approval of this research<br>• Indicate the form of consent obtained (written/oral) or the reason that consent was not obtained (e.g. the data were analyzed anonymously)<br><br>**Animal Research (involving vertebrate animals, embryos or tissues)**<br>• Provide the name of the Institutional Animal Care and Use Committee (IACUC) or other relevant ethics board that reviewed the study protocol, and indicate whether they approved this research or granted a formal waiver of ethical approval<br>• Include an approval number if one was obtained<br>• If the study involved *non-human primates*, add *additional details* about animal welfare and steps taken to ameliorate suffering<br>• If anesthesia, euthanasia, or any kind of animal sacrifice is part of the study, include briefly which substances and/or methods were applied<br><br>**Field Research**<br><br>Include the following details if this study involves the collection of plant, animal, or other materials from a natural setting:<br>• Field permit number<br>• Name of the institution or relevant body that granted permission	
**Data Availability**<br><br>Authors are required to make all data underlying the findings described fully available, without restriction, and from the time of publication. PLOS allows rare exceptions to address legal and ethical concerns. See the PLOS Data Policy and FAQ for detailed information.	Yes - all data are fully available without restriction

A Data Availability Statement describing where the data can be found is required at submission. Your answers to this question constitute the Data Availability Statement and **will be published in the article**, if accepted.<br><br>**Important:** Stating 'data available on request from the author' is not sufficient. If your data are only available upon request, select 'No' for the first question and explain your exceptional situation in the text box.<br><br>Do the authors confirm that all data underlying the findings described in their manuscript are fully available without restriction?	
**Describe where the data may be found in full sentences. If you are copying our sample text, replace any instances of XXX with the appropriate details.**<br><br>• If the data are **held or will be held in a public repository**, include URLs, accession numbers or DOIs. If this information will only be available after acceptance, indicate this by ticking the box below. For example: *All XXX files are available from the XXX database (accession number(s) XXX, XXX.).*<br>• If the data are all contained **within the manuscript and/or Supporting Information files**, enter the following: *All relevant data are within the manuscript and its Supporting Information files.*<br>• If neither of these applies but you are able to provide **details of access elsewhere**, with or without limitations, please do so. For example:<br><br>*Data cannot be shared publicly because of [XXX]. Data are available from the XXX Institutional Data Access / Ethics Committee (contact via XXX) for researchers who meet the criteria for access to confidential data.*<br><br>*The data underlying the results presented in the study are available from (include the name of the third party*	All relevant data are within the manuscript and its Supporting Information files.

and contact information or URL).<br>• This text is appropriate if the data are owned by a third party and authors do not have permission to share the data.<br><br>* typeset	
Additional data availability information:	

Manuscript
Click here to access/download;Manuscript;Manuscript.docx

Towards Resilience: Transcriptional Insights on Flavonoid Biosynthesis During Peanut Seed Maturation Phases

Gustavo Roberto Fonseca de Oliveira[1☯*]; Liam Walker[2‡]; Rômulo Pedro Macêdo Lima[1‡]; Thiago Barbosa Batista[3‡]; Clissia Barboza Mastrangelo[3‡]; Edvaldo Aparecido Amaral da Silva[1☯]

[1]School of Agronomy Science, São Paulo State University, Botucatu, São Paulo, 18610-034, Brazil.
[2]School of Life Sciences, University of Warwick, Coventry, CV4 7AL, UK.
[3]Laboratory of Radiobiology and Environment, Center for Nuclear Energy in Agriculture, University of São Paulo, Piracicaba, São Paulo, 13416-000, Brazil.

* Corresponding author:
E-mail: grfonseca.agro@gmail.com
☯These authors contributed equally to this work
‡These authors also contributed equally to this work

Abstract

Flavonoids are secondary metabolites widely studied as a metabolic protector against several stressors in plants, yet they are understudied in the events of peanut seed development. Substantial advances in the understanding of peanut seed maturation have been made in recent years, however, the role of flavonoids in this process is unclear. Here, the fundamental question asked was: are flavonoids involved in peanut seed maturation phases? This study investigates whether the main transcripts associated with the flavonoid pathway, such as anthocyanins, are biologically linked to the physiological quality components of peanut seeds during development. For this purpose, peanut seeds classified into five stages (R5, R6, R7, R8 and R9) ~~which~~ were used for the evaluation of quality attributes, such as desiccation tolerance, vigor and longevity parameters, and for RNA sequencing (RNAseq). Interestingly, anthocyanins accumulated more in the beginning of the seed filling phase, coinciding with the upregulation in RNAseq and quantitative PCR of key genes in its pathway, such as *AhCHS* (*0FI6RG*), *AhCHI* (*VJQ7J1*), *AhFLS* (*4Y1607*), *AhLDOX* (*AQ6B1J*) and *AhANR* (*IK60LM*). Additionally, we found that *AhMYB12* (*PR7AYB*) and *AhMYB308* (*TG6F30*) exhibited increased expression at the early stages (R5 and R6) and decreased at the later ones (R7, R8 and R9)~~,~~ ~~respectively~~. *AhCHS* expression acts in synergy with well-known seed maturation regulators, such as

the ABA response (e.g., ABI5 and ABI3). The involvement of flavonoid biosynthesis in peanut seed development is suggested here as a contributor to its resilience during the acquisition of physiological quality attributes, highlighting the molecular aspects associated with survival in the dry state.

Key words: *Arachis hypogaea* L., anthocyanin, MYB, chalcone, seed development

Introduction

Flavonoids comprise an important group of secondary metabolites with an antioxidant role that has been broadly investigated in plants [1,2]. These compounds are generally present in the cell vacuole and they are widely found in structures such as leaves, flowers, fruits and seeds [3]. Depending on the plant species and organ, different types of flavonoids can help in combating pathogens, insect attacks and excess free radicals [4,5]. Anthocyanin, in addition to these functions, has been described as a pigmenting flavonoid that mitigates oxidative stress in plant tissues, ensuring cellular homeostasis under biotic and abiotic adversities [6,7]. In dry seeds, anthocyanin participates in an autoimmune system that reacts to aging and exposure to thermal stress, with protective effects that extend to the seedlings [8,9]. This represents an interesting embryonic resilience strategy that, like many others, is generally acquired during seed development and is associated with physiological changes and established molecular mechanisms [10,11]. Considering this, we investigated whether the transcription dynamics of flavonoids, such as anthocyanin, could be involved in with the acquisition of seed quality attributes during the maturation phases.

Seeds complete their development in an ordered maturation program subdivided into a time series marked by seed filling/maturation and what we recognize nowadays as late maturation [12,13]. The maturation phase of desiccation-tolerant seeds (orthodox) is primarily characterized by abrupt changes in water status and weight [14,15]. The seeds undergo a constant accumulation of reserves components such as proteins, sugars and oil, in addition to high hormonal/metabolic activity and molecular regulation [16–18]. It is an “orchestra” of agents that work to ensure the preservation of seed life during desiccation and storage [19,20]. In the late maturation phase, the accumulation of

reserves is completed and the seeds continue to lose water and consolidate cellular stability towards the acquisition of vigor and longevity [12,13]. These components allow the seeds to do something extraordinary in nature: germinate and generate seedlings when hydrated after a prolonged dry state [11,21]. This is extremely important for agriculture and food security, since seeds harvested in one season need these physiological resources to generate high-performance plants in the following production cycle [22–24]. Therefore, the fundamental question to be answered is: what is the collaborative part of flavonoids, such as anthocyanin, in this "orchestra" programmed for the acquisition of components necessary to survive the dry state? Flavonoids are widely studied in the context of plant antioxidant defense but the potential contribution of flavonoids in the context of maturation phases has been very little discussed [5,25,26]. This is even less investigated in peanuts, an oilseed of great relevance for global agriculture [27,28].

The flavonoid biosynthesis pathway is widely known [1,4]. The main associated enzymes include chalcone synthase (CHS), chalcone isomerase (CHI), flavonol synthase (FLS), leucoanthocyanidin dioxygenase (LDOX) and anthocyanidin reductase (ANR) [5,29]. Additionally, proteins of the MYB family transcription factors often activate or repress the expression of the flavonoids genes [30,31]. For example, MYB12 directly induces the synthesis of different flavonoids according to the biological demand for protection in plants [32,33]. On the other hand, MYB308 represses this synthesis but enriches other agents of the plant secondary metabolism pathways in return, modulating enhanced self-immunity [34–36]. Therefore, these transcription factors regulate the expression of genes from a "powerful toolkit" associated with protection [34–36]. Interestingly, other key players associated with seed maturation phases have a similar protective role in the molecular level [10,21]. Several heat shock proteins (HSPs), late embryogenesis abundant proteins (LEAs) and oligosaccharides from the raffinose family (RFOs) coordinate the protection of seeds during desiccation and the acquisition of longevity [10,16,37]. In particular, proteins with chaperone activity, such as HSP70, play a key role in guiding other proteins to fold correctly into their functional three-dimensional shapes, ensuring that they operate efficiently, especially during stress conditions

[38,39]. These events are also regulated by transcription factors such as those associated with the ABA response (e.g., ABI5 and ABI3), which have crucial functions in seed maturation [12,15,40,41]. Thus, we explored the possible links between flavonoids and these coordinators of peanut seed development. The hypothesis tested was that flavonoids are transcriptionally involved in peanut seed development, acting systematically on secondary metabolism throughout the maturation phases.

Material and Methods

Seed Samples

Peanut seeds of the Runner type belonging to the cultivar IAC OL3 were used. The seeds were produced at the Experimental Farm Lageado of São Paulo State University, Botucatu, São Paulo, Brazil, in the 2021/2022 season. The material was chosen considering its high yield and significant adoption by Brazilian farmers. The field conditions for crop management, including soil preparation, fertilization (top dressing) and weather variations during the plant cycle (rainfall, temperature and relative humidity, RH), were described (2021/2022 crop season) [24]. Additionally, following the methods presented by those authors, the seeds were collected in the field, classified according to their development stages (R5, R6, R7, R8 and R9) and a portion of them was dried until it achieved 10% of water content. The samples were used to conduct physiological assessments. For the molecular analyses, such as RNA sequencing (RNAseq) and quantitative PCR (qPCR), we use fresh seeds immediately frozen after harvesting. The details of which are presented below.

Seed Physiology and Anthocyanin

Water and Weight

The water determination took place by using five replicates of ten fresh seeds (n=50) from each development stage. The material was put into a cylinder container with a lid and weighed on a precision balance (accurate to 0.0001 g). The seeds were kept at 105ºC for 24 hours (oven method) [42]. The water content was expressed in humidity degree on a wet basis (%). For the dry weight

determination, another five replicates of ten seeds were used (n=50). Similar containers were adopted and the seeds were kept at 60ºC for 72 hours. The dry weight was expressed in grams per 10 seeds [24].

Desiccation Tolerance

Five replicates of 25 dry seeds from each development stage were used (n=125). The method for peanut seed coat removal and sample sterilization (1% hypochlorite) was followed as described [24]. The seeds were placed on paper rolls moistened with deionized water (1:2.5, g:mL). The material was put in an incubator set to 20ºC for 12 hours (lights off) and 30ºC for another 12 hours (lights on), as described [43]. The desiccation tolerance (%) was assessed 10 days afterwards, considering radicle protrusion ≥ 2 mm as germinated seed criterium.

Time to 50% Germination (t50)

For the t50 test, five replicates of 25 dry seeds were used (n=125). The procedures to promote seed germination described above were adopted as well [24]. Germination, expressed by radicle protrusion ≥ 2 mm, was evaluated every 4 hours from the start of the test. The software Germinator was used to calculate the t50 according to the seed stages [44]. The t50 was expressed in hours, where more time to reach 50% germination indicates lower seed performance.

Seedling Assessments

For the analysis of seed vigor through seedling performance, five replicates of 10 dry seeds from each development stage were used (n=50). The seeds were placed in paper rolls, in the upper third, with the hilum facing downwards. The paper was moistened as previously (1:2.5, g: mL) and they were placed in a controlled incubator (20ºC for 12 hours with no light/ 30ºC for 12 hours with light). After 7 days, the total length of the seedlings was measured (cm). Additionally, the shoots and roots were separated to determine the dry weight, expressed in milligrams (60ºC for 72 hours) [24].

Germination of Aged Seeds

For each development stage, 145 dry seeds were subjected to the aging method [45]. The seeds were placed on a wire mesh, set inside a plastic box containing 40 mL of water and kept at 42ºC for 3 days. After aging, seed coat removal and sterilization procedures were adopted, as described [24]. Germination was performed using five replicates of 25 seeds (n=125). Two replicates of 10 seeds were used for the water content determination, as previously described. The germination of aged seeds was obtained after 5 days, considering radicle protrusion ≥ 2 mm as criterium.

Seedling Emergence

Five replicates of 25 dry seeds from each development stage were used (n=125). The seeds were sown in a sand compost equidistantly and under field conditions. The sand was watered to 60% of its retention capacity. The weather variables were monitored during the test as described [24]. The criterion for counting seedling emergence (%) was the appearance of shoots above the sand with expanded leaves by the 21st day. The speed index was calculated based on the sum of daily counts of seedlings over time (days), as described [46,47].

Plant Establishment in the Field and Shoot Dry Weight

Five replicates of 20 dry seeds (n=100), stored for 6 months (10ºC and 55% RH), were used. The seeds were sown in the soil in five parallel lines for each development stage (1m, 90 cm apart), one line per replicate. No chemical treatments were applied on the seeds so that their natural self-defenses under field conditions could be evaluated. Water supplementation was necessary (15 mm, sprinkling) to ensure basic moisture for seed germination. Plant establishment (%) was counted on the 30th day after sowing. Finally, all the plants in the lines (plant stand) had their shoots cut off for dry weight assessment (60ºC for 72 hours, milligrams).

Germination of Stored Seeds

To check their storage life, 145 dry seeds at each development stage were used. The seeds were arranged on a piece of gauze cloth, which was fastened to the top surface of a container, as described [24]. The container's bottom was filled with a saturated NaCl solution. After sealing the box tightly, it was placed in an incubator at 35°C and 75% RH for 100 days. Germination was performed using five replicates of 25 seeds (n=125). Additionally, water content was determined (around 7%) using two replicates of 10 seeds, as described previously. Radicle protrusion ≥ 2 mm was considered as the criterion for germination (%)10 days after the beginning of the test. Seedlings with well-formed shoots, fully developed roots and total length ≥ 3 cm were considered normal (%). The results expressed the capacity of the seeds to tolerate storage, keeping their viability under stress conditions (high temperature and relative humidity).

Anthocyanin and Multispectral Images

Five replicates of 15 dry seeds from each development stage were used (n=75). Multispectral images were captured non-destructively with a SeedReporter™ (PhenoVation, Netherlands) to calculate the anthocyanin index, as described [9]. Light intensity was adjusted to prevent overload and reflectance images (2448 × 2448 pixels, 3.69 µm/pixel) were acquired under broad-band white light (3000 K, 450–780 nm) using filters at 540, 710 and 770 nm. The anthocyanin index was computed with SeedReporter™ software (v5.5.1) following described procedures [48].

Seed Molecular Assessments

RNA Extraction

Three replicates of 15 fresh seeds (n=45) were frozen in liquid N immediately after they had been collected at each development stage. Seed samples were stored in a deep freezer at -80ºC. Each replicate was manually crushed in a crucible with a pestle until it reached a powdery appearance. This procedure was always performed in the presence of liquid N. The crushed material was placed into

1.5 mL tubes and stored at -80ºC, as described [49]. For RNA extraction, the NucleoSpin RNA Plant® Kit from Macherey-Nagel was used according to the manufacturer's instructions. RNA purity was determined by UV spectrophotometry (260 nm, NanoDrop 1000, Thermo Fischer Scientific) and its integrity was determined using a Bioanalyzer, Agilent Technologies (Life Sciences Core Facility, LaCTAD, State University of Campinas, UNICAMP) (https://www.lactad.unicamp.br/). Only samples with an RNA Integrity Number (RIN) > 7 were used for RNA sequencing (RNAseq), as shown in the supplemental material (S1 Table).

RNAseq and Bioinformatics

The sequenced RNAs (illumina platform, HiScan; 150 bp paired-end and with a number of lanes equal to two), mean scores ≥ 20 per position were used. Raw reads were trimmed using fastP [50] with the following parameters; cut_right, cut_window_size=4, cut_mean_quality=20 and length_required=40. Alignment of trimmed, clean reads and quantification of counts per gene was conducted using STAR [51] with the sjdbOverhang parameter set to 149 (equal to read length -1). The index files for STAR were generated using the *Arachis hypogaea* accession (arahy.Tifrunner.gnm1.ann1.CCJH.gene_models_main.gff3.gz) Tifrunner annotation files from genome assembly 1.0 [52]. Mapping reads of the peanut genome are available in supplemental information (S2 Table) and the less recent annotation file from genome assembly 1.0 was chosen due to compatibility with the Phytozome database [53] (https://phytozome-next.jgi.doe.gov/). Differentially expressed genes were obtained using DESeq2 [54]. Changes in gene expression were considered statistically significant when Log_2 Fold Change (LFC) was ≤ -2 and ≥ 2. The differentially expressed genes (DEGs) were obtained with adjusted *p*-values cutoff ≤ 0.001. The key molecular players of seed maturation phases were highlighted in the DEGs data considering previous studies [10,11,15,16].

Gene Ontology (GO) and ortholog identification

The GO enrichment analysis was carried out through the platform agriGO v2.0 [55]. Significantly differentially expressed genes were queried for overrepresented ontologies against the *Arachis hypogaea* cv Tifrunner (*Ah*) database (http://systemsbiology.cau.edu.cn/agriGOv2/). Amongst the most enriched processes was flavonoid biosynthesis which was identified with a false discovery rate (FDR) < 0.000001. To identify orthologs of characterized flavonoid biosynthesis genes from other species [6,32], the protein homologs tool of Phytozome 13 (https://phytozome-next.jgi.doe.gov/) and NCBI BLAST (https://blast.ncbi.nlm.nih.gov/Blast.cgi) were used. The normalized gene count data (DESeq2, rlog) was then used to obtain the expression of these genes from our data.

RT-qPCR Study

To validate the differentially expressed genes associated with anthocyanin synthesis identified in the RNAseq, an RT-qPCR study was performed. For this, primers for the target genes were designed (Table S3) based on the nucleotide sequences of the corresponding transcripts, using the Primer 3 platform (https://primer3.ut.ee/) [56]. The same total RNA sequenced in three replicates for each development stage (R5, R6, R7, R8 and R9) was used. cDNA synthesis was performed using the High-Capacity cDNA Reverse Transcription Kit (Thermo Fisher). The amplification protocol consisted of 3 minutes at 95ºC, followed by 40 cycles of 15 seconds at 95ºC and 1 minute at 60ºC and a final step of 1 minute at 95ºC and 1 minute at 55ºC. The $2^{-\Delta\Delta Ct}$ method was used [57], where ΔΔCt represents the normalized expression of the target gene against the reference gene, 60S ribosomal protein L10 described in peanut studies [58,59]. The expression of the genes during seed development was calculated relative to a pool of all seed stages.

Data Analysis

The experiment involved randomly assigning five development stages (R5, R6, R7, R8 and R9) as treatment groups, with five independent replicates for each stage (n=125). Mean differences among the groups were determined through the Scott-Knott test, applying a significance threshold of

p value ≤ 0.05. Additionally, The PCA made with seed physiology and anthocyanin data was presented with a PERMANOVA test performed using Canoco 5 software [9,60]. Correlation analyses were carried out using the Spearman method. Finally, Mean Decrease Gini (MDG) was obtained with a random forest algorithm, where variables with higher MDG values are key genes, which are the predictors of seed development stages [24]. The data analysis was performed using R software [61].

Results

Physiological Quality and Anthocyanin in Peanuts

The basic events of developing seeds followed patterns with sequential logic; water content decreased between stages R5 and R9 (70-30%), coinciding with an increase in dry weight (Fig 1A-B). The filling phase occurred up to R7, the stage at which the seed began to tolerate desiccation and express viability in the dry state (63.2%) (Fig 1C). Even with stable weight (7.58 g/10 seeds), the seeds continuously enhanced their capacity to germinate quickly and establish as seedlings after storage (Fig 1D-N). This phase marked the late maturation, where indicators such as vigor (Fig 1F-G, I-J) and longevity (Fig 1K-N) were optimized until the seed reached full maturity at stage R9.

Curiously, the anthocyanin results were entirely opposite (PC_1: 87.3%) to the acquisition of seed quality attributes during development (Fig 1 and Fig 2A). The results revealed a remarkably ordered pattern indicating differentiated levels of anthocyanin in dry seeds according to the maturation stages (Fig 2A). The anthocyanin index was high in the early stages (R5 and R6) and decreased significantly as the seed advanced to the late ones (R7, R8 and R9) (Fig 2B). This behavior coincided with the observed natural dehydration (70-30%) and resulted in a positive correlation (0.87) between anthocyanin and water content (Fig 2C). All other variables such as dry weight, desiccation tolerance, t50, seedling length, shoot and root dry weight of seedlings, germination of aged seeds, seedling emergence speed, seedling emergence, plant establishment in the field, shoot dry weight of plant stand, germination of stored seeds and normal seedlings of stored seeds showed a negative correlation with anthocyanin levels (ranging from -0.92 to -0.77). Therefore, anthocyanin abundance

is strongly associated with the early stages of seed development (filling phase) and negatively correlates with traits associated with seed longevity (Figs 1 and 2).

RNAseq and Flavonoid Biosynthesis

To identify molecular mechanisms underpinning the anthocyanin dynamics during maturation phases, we performed RNAseq analysis (see methods) of all developmental stages of peanut seeds. Principal component analysis (Fig 3A) showed separation between the early (R5 and R6) and late seed stages (R7, R8 and R9). The variability associated with principal component 1 of the transcriptome data (Fig 3A; PC_1: 91%) was similar to that obtained from seed physiology (Fig 2A; PC_1: 87.3%), demonstrating both these domains vary significantly during peanut seed development. Based on this result, differentially expressed genes were calculated between the early and late seed stages providing 8,854 significantly differentially expressed genes, of which 7,028 were upregulated and 1,826 were downregulated between the early and late stages, respectively (Fig 3B). The list of significant genes associated with the RNAseq component of this work is fully available in the supplementary material (S4 File).

We then queried our DEGs for overrepresented biological activities using gene ontology. Significantly enriched biological processes included cell wall formation, lipid biosynthesis, chlorophyll biosynthesis and hormone regulation (Fig 3C). In particular, one of the most enriched processes was flavonoid biosynthesis, (GO: 0009813) highlighting the moderation of anthocyanin biosynthesis (Fig 3C). Higher gene expression was observed for a chalcone isomerase (*AhCHI*), chalcone synthase (*AhCHS*), flavonol synthase (*AhFLS*), anthocyanidin reductase (*AhANR*) and leucoanthocyanidin reductase (*AhLOX*) at stages R5 and R6. The transcription factor (TF) *AhMYB12* exhibited increased expression in the early stages and reduced expression in the late stages. Conversely, TF *AhMYB308* was only highly expressed in the late maturation stages (Fig 3D).

RT-qPCR: Validation of Genes During Seed Development

We identified increased expression in immature seeds (R5 and R6) of key genes involved in flavonoid biosynthesis throughout the different parts of the pathway: the beginning (*AhCHS* and *AhCHI*), middle (*AhFLS*) and end (*AhLOX* and *AhANR*). We validated the expression of these genes using qPCR and confirmed their relative expression (ΔΔCt) was significantly higher at the early stages of seed development (Fig 4A-E). The transcription factors *AhMYB12* and *AhMYB308* were identified as potential regulators, as an activator ("on") and repressor ("off") respectively, of these flavonoid genes (Fig 4). Expression of *AhMYB12* was also higher at R5 and R6 (Fig 4F) whilst the expression values of *AhMYB308* were quite low at these stages and higher at later ones with relative expression peak~~ing~~ at R7 (Fig 4G). Finally, *AhCHS* was classified, based on Mean Decrease Gini, as the most relevant gene for predicting peanut seed development (Fig 4H).

Genes associated with secondary metabolism in late maturation

In the GO analysis, within the flavonoid biosynthesis significant processes, a total of 14 downregulated genes were found. This negative regulation means that these genes were more expressed in the late stages (R7, R8 and R9). They were: UDP-glycosyltransferase 83A1, stilbene synthase 3-like, crocetin glucosyltransferase, beta-glucosidase 10, beta-glucosidase 11, hydroxyisourate hydrolase, pathogen-inducible salicylic acid glucosyltransferase, putative UDP-rhamnose:rhamnosyltransferase 1, linamarin synthase 2, putative stilbene synthase 2, Senescence-Related Gene 1, cryptochrome-1, vestitone reductase and UDP-glycosyltransferase 71K2. Interestingly, all of these had some documented relationship to the secondary metabolism (Table 1), which indicated a defensive protective strategy as the seed progressed towards late maturation.

Key Players in Seed Maturation Phases

Due to our results suggesting that expression levels of *AhCHS* was the most important predictor of seed development (Fig 4H), the link between expression of *AhCHS* and other key players, as determined by literature review, involved in seed maturation (Table 2) was established in our

RNAseq data by correlation analysis (Fig 5). At early stages, correlations ranging from 0.73 to 0.95 were found between *AhCHS* and the following groups: 1) abscisic acid response (*ABI5* and *ABI3*); 2) B3 family (*REM23*, *FUS3* and *DOG1*); 3) gibberellin and auxin (*GA20ox1* and *AGR2*); 4) LEA proteins (*LEA3* and *LEA*2); 5) raffinose family oligosaccharide (*GSGT5*); and 6) heat shock proteins (*HSP18*, *HSFA2*, *HSP70* and *HSP21*). The similar transcriptional dynamics of *AhCHS* to these genes indicates that their upregulation is also prioritized in the seed filling phase (early stages). In contrast, at later stages, *AhCHS* expression correlations ranging from -0.63 to -0.84 were identified in the following groups: 7) LEA proteins (*LEA5*, *LEA14*, *LEA29* and *LEA34*); 8) heat shock proteins (*HSP19*, *HSP27* and *HSP70*); 9) sugar metabolism and transport (*GSGT*, *SPS2*, *SWEET6b* and *STP10*); 10) phenylpropanoid biosynthesis (*PAL*); and 11) antioxidant enzymes (*STS3* and *GR*). Clearly, other relevant genes showed prominent regulation during the late maturation phase, when *AhCHS* expression was minimized and seeds acquired the ability to survive in the dry state (Fig 5A-B).

Discussion

Linking Anthocyanin Dynamics with Peanut Seed Development

Anthocyanin levels differ according to the seed water dynamics (Fig 2C). It was observed that high water content is favorable for anthocyanin storage in cell vacuoles and, upon dehydration, this accumulation is maintained in the dry weight of the tissue (Fig 2B) [62,63]. During orthodox seed maturation, humidity conditions of 70–65% are commonly found during the early stages (Fig. 1A). These stages are characterized by intense cellular changes, such as cell division, which occur under high water demand [64–66]. Additionally, seed filling is also intense in the early stages, where the high metabolic activity (i.e., respiration) is capable of generating excess free radicals that cause damage to cells [15,67]. Anthocyanins could adjust toxic oxidative levels, contributing to the deposition of reserves within a metabolically balanced framework of the seed [5,68–70]. Conversely, at the late stages there are agents, such as LEAs and HSPs, with functions that extend beyond the

capacity of flavonoids (Fig. 5A). One example is the stabilization of proteins and cellular membranes at the molecular level under lower seed water content (45–30%) [16,71,72]. Probably because of this, the anthocyanin enrichment that is maintained at the beginning of maturation is less required at the end of seed development (Fig 2B). It is reasonable to suggest that flavonoid regulation appears to prepare the most immature seeds to acquire an appropriate chemical composition (Fig 1B), while gradually tolerating desiccation (Fig 1C). The crucial reserves such as proteins (LEAs and HSPs), sugars (ROFs), oil and minerals (K and Ca) are essential for cellular self-defense under seed water changes towards the late stages [43,69]. This could mitigate potential cellular stressors, acting as a "metabolic guard" so that the maturation phase (seed filling) and the shift to late maturation may occur under homeostasis [24].

Flavonoid Biosynthesis: Transcriptional Changes Throughout Seed Development

The enriched flavonoid biosynthesis in our GO analysis (Fig 3C) highlights the existence of transcriptional programs that may modulate defense mechanisms throughout peanut maturation [73–75]. Interestingly, seed anthocyanin accumulation was aligned with the upregulation of key genes in its biosynthesis pathway, suggesting that both events were biologically correlated (Fig. 2B, 3D). *AhCHS* was the key gene identified during seed development (Fig. 4H), centrally positioned as a "bridge" in the pathway that produces other flavonoids involved in plant resistance [76,77]. It is important to note that this pathway is also mediated by induction/repression "switches" within the pathway (Fig 5) [31,33,36]; for instance, the transcription factor *AhMYB12* is described as performing an essential role in flavonoid biosynthesis by positively regulating associated gene expression (Fig 3D) [32,33]. Indeed, its increased activity coinciding with increased gene expression suggests its transcriptional demand was required in early seed stages (Fig 3D) [30,31]. Curiously, in the late stages, most flavonoid genes showed decreased expression levels, while *AhMYB308* exhibited increased transcription (Fig. 4G). It's possible that it represses key flavonoid biosynthetic genes (Fig. 3D) while increasing the availability of other precursors of secondary metabolism [32,34]. This

flexible action of *AhMYB308* may contribute to the establishment of a “stress protection toolkit” in the late stages (Table 1). The protection mechanisms in peanuts appear to function as a dynamic network of nuclear decisions, regulating transcriptional changes based on the demands of each maturation phase (Fig 3 and 4).

The “Toolkit” for Seed Resilience: Exploring Secondary Metabolism During Late Maturation

We identified a set of genes upregulated exclusively during the late stages of peanut seed development (Table 1) which we hypothesize may equip the developing seed with a “toolkit” to ensure it is prepared for germinating and subsequent establishment [4,10,78]. The genes found include those associated with antioxidants, antifungals, cyanogen common in other species (e.g., *Manihot esculenta*), photoreceptors associated with seed dormancy, thermoprotectants, N metabolism contributors, cell wall structural components (e.g., lignin, pectin and suberin), insecticidal compounds and stress tolerance agents, which have all been documented (Table 1) [26,79]. An interesting aspect to this is that the implementation of this strategy takes place in an underground environment where the diversity of biotic and abiotic factors that can interfere in the seed development is vast [80,81]. Perhaps this singular habit of maturation under these potential stressors justifies the great embryonic protection established transcriptionally (Fig 3C). Furthermore, an important aspect of this protective enhancement is the seed's chemical composition, which is predominantly made up of lipids (48–50%), prone to oxidation [15,43,82]. Unsaturated fatty acids, such as linoleic acid, are significant sources of peroxidation, which demand robust enzymatic performance from the cellular machinery to protect it from lipid instability [83,84]. Thus, the peanut development appears to have evolved with a reinforcement of genetic configurations that provide robust support for the viability of its offspring. This enhanced late secondary metabolism may function as a sophisticated “toolkit”, providing necessary resilience to: i) both biotic and abiotic stresses; ii) acquisition of dry-state survival; and iii) maximum protection during peanut seed storage life (Fig 1M-N).

Key Players in Peanut Seed Development: The Birth of Longevity in the Dry State

We identified distinct groups of genes that are preferentially expressed during the different stages of the peanut seed's journey towards full maturity (Table 2). Flavonoid biosynthesis appears to be one of the gears in a sophisticated set of mechanisms that contribute to the molecular events required for proper seed development (Fig 5) [85,86]. Its workflow in the beginning of peanut maturation coincides with the activity of master orchestrators of legume seed development (Fig 5A), such as ABA responses described in model plants [12,40]. These key players are associated with acquisition of desiccation tolerance, longevity and modulation of flavonoid biosynthesis through the MYB family of transcription factors [40,87,88]. Notably, flavonoids positively correlate with a diverse set of genes and transcription factors (e.g., *FUS3* and *HSFA2*) extending to regulators of lipid and carbohydrate synthesis, agents of hormonal balance, seed dormancy and chaperone protein synthesis (Fig 5, Table 2) [16,38,39,72,89]. It is possible that the activities of these genes are coordinated in synergy with the upregulation of anthocyanin pathway genes (Fig. 4), as part of a shared natural effort to acquire seed quiescence [18,19].

In late maturation. other agents such as chaperone proteins (e.g., HSP70), sugar transporters, phenylpropanoids and antioxidants were genetically orchestrated (Table 2) [78,90–92]. These protective mechanisms have been described as promoters of molecular stability throughout seed storage time, preserving vigor and ensuring the continuity of the species across different environmental scenarios [10,11,13,93]. What is interesting to consider is how peanut seeds are genetically prepared to survive in nature only if they experience late maturation, a phase in which maximum physiological quality is acquired (Fig 1) [11,24,94]. Flavonoid biosynthesis results from a network of transcriptional decisions in the cell nucleus, which could thus be considered to be the birthplace of seed storage life (Fig 5B). Thus, the correlation of flavonoid synthesis with the long survival of seeds in the dry state can be considered part of the path towards full peanut maturity (stage R9).

Applications in Agriculture and Future Approaches

Our work highlights some findings that may be of interest in an agricultural setting. The first is the use of technologies, based on multispectral images to detect seeds from the late stages by analyzing anthocyanin levels non-destructively, as a strategy for mitigating the uneven maturation inherent in peanut plants [24]. This could optimize the selection of seeds with superior longevity and enhanced bioprotection [8,9], since an increase in anthocyanin reflects embryonic immaturity during maturation phases (Fig 1-2). In the cellular context, assessing the expression levels of *AhCHS* (would allow the evaluation of seed maturity as a proxy of the extent of flavonoid biosynthesis (Fig 3) [24,76,77]. This could become a potential tool for seed samples analysis, enabling real-time results through qPCR, adapted to industrial demands (Fig 4). Additionally, our findings are valuable for initiating classical plant breeding programs aimed at selecting peanut lines through molecular markers linked to the secondary metabolism (Table 1). This approach could lead to new varieties with enhanced resilience, improving resistance against different kinds of stresses [4–7]. From the food industry's perspective, new peanut-based products enriched with antioxidants could be developed, highlighting the nutritional benefits for human health [95]. Regarding future approaches, gene-editing using CRISPR-Cas9 could help refine our understanding of the mechanisms associated with the genes we identified (Fig 4) [96]. These results provide may be useful for the biotechnological development of new peanut crops (Table 2), especially in future attempts to address challenges posed by extreme climate changes [97]. Thus, these findings can contribute to technological steps towards the constant improvement of the peanut chain in producing nations.

Conclusions

The peanut has a unique physiology and biology among cultivated legume species. The involvement of flavonoid biosynthesis in seed development is suggested here as a contributor to its resilience during the acquisition of physiological quality attributes. The associated transcriptional changes implement secondary metabolism responses adapted to each maturation phase towards full

seed maturity. We present a potential mechanism in peanut that contributes to seed survival in the dry state, with maximum storage life and enhanced bioprotection.

Supporting information

S1 Table. Quality control of RNA samples extracted from fresh peanut seeds.

S2 Table. Reads mapped to the peanut genome.

S3 Table. Primers designed for RT-qPCR study.

S4 File. Significant genes associated with the RNAseq.

Acknowledgements

We are thankful to Roger Hutchings for the English review of the manuscript. We also thank the University of Warwick (School of Life Sciences, UK) and Professor George Bassel for their valuable support throughout the bioinformatics analysis. The authors G.R. Fonseca de Oliveira and C.B. Mastrangelo are also grateful to the National Council for Scientific and Technological Development (CNPq), grant nº 142236/2020-9 and grant nº 314305/2021-1, respectively.

Funding

This study was funded by São Paulo Research Foundation – FAPESP grant number #2020/14050-3 and #2023/00435-9 for G.R. Fonseca de Oliveira, #2021/07331 for E.A. Amaral da Silva, and #2017/15220-7 and #2018/03793-5 for C.B. Mastrangelo.

References

1. Shah A, Smith DL. Flavonoids in agriculture: Chemistry and roles in, biotic and abiotic stress responses, and microbial associations. Agronomy. 2020; 10: 1-26. https://doi:10.3390/agronomy10081209
2. Dias MC, Pinto DCGA, Silva AMS. Plant flavonoids: Chemical characteristics and biological

activity. Molecules. 2021; 26: 1–16. https://doi:10.3390/molecules26175377

3. Shen N, Wang T, Gan Q, Liu S, Wang L, Jin B. Plant flavonoids: Classification, distribution, biosynthesis, and antioxidant activity. Food Chem. 2022; 383: 1-13. https://doi:10.1016/j.foodchem.2022.132531

4. Zhuang W, Li Y, Shu X, Pu Y, Wang X-J, Wang T, et al. The Classification, Molecular Structure and Biological Biosynthesis of Flavonoids, and Their Roles in Biotic and Abiotic Stresses. Molecules. 2023; 28: 1-26. https://doi:10.3390/molecules28083599

5. Shirley BW. Flavonoids in seeds and grains: Physiological function, agronomic importance and the genetics of biosynthesis. Seed Sci Res. 1998; 8: 415–422. https://doi:10.1017/s0960258500004372

6. Yan W, Li J, Lin X, Wang L, Yang X, Xia X, et al. Changes in plant anthocyanin levels in response to abiotic stresses: a meta-analysis. Plant Biotechnol Rep. 2022; 16: 497–508. https://doi:10.1007/s11816-022-00777-7

7. Cappellini F, Marinelli A, Toccaceli M, Tonelli C, Petroni K. Anthocyanins: From Mechanisms of Regulation in Plants to Health Benefits in Foods. Front Plant Sci. 2021; 12: 1–23. https://doi:10.3389/fpls.2021.748049

8. Sudki JM, Fonseca de Oliveira GR, de Medeiros AD, Mastrangelo T, Arthur V, Amaral da Silva EA, et al. Fungal identification in peanuts seeds through multispectral images: Technological advances to enhance sanitary quality. Front Plant Sci. 2023; 14: 1–11. https://doi:10.3389/fpls.2023.1112916

9. Fonseca de Oliveira GR, Mastrangelo CB, Hirai WY, Batista TB, Sudki JM, Petronilio ACP, et al. An Approach Using Emerging Optical Technologies and Artificial Intelligence Brings New Markers to Evaluate Peanut Seed Quality. Front Plant Sci. 2022; 13: 1–18. https://doi:10.3389/fpls.2022.849986

10. Lima JJP, Buitink J, Lalanne D, Rossi RF, Pelletier S, Da Silva EAA, et al. Molecular characterization of the acquisition of longevity during seed maturation in soybean. PLoS One.

2017; 12: 1–25. https://doi:10.1371/journal.pone.0180282

11. Leprince O, Pellizzaro A, Berriri S, Buitink J. Late seed maturation: Drying without dying. J Exp Bot. 2017; 68: 827–841. https://doi:10.1093/jxb/erw363

12. Zinsmeister J, Lalanne D, Ly Vu B, Schoefs B, Marchand J, Dang TT, et al. ABSCISIC ACID INSENSITIVE 4 coordinates eoplast formation to ensure acquisition of seed longevity during maturation in Medicago truncatula. Plant J. 2023; 113: 934–953. https://doi:10.1111/tpj.16091

13. Buitink J, Leprince O. Intracellular glasses and seed survival in the dry state. Comptes Rendus - Biol. 2008; 331: 788–795. https://doi:10.1016/j.crvi.2008.08.002

14. Costa MCD, Cooper K, Hilhorst HWM, Farrant JM. Orthodox seeds and resurrection plants: Two of a kind? Plant Physiol. 2017; 175: 589–599. https://doi:10.1104/pp.17.00760

15. Bewley JD., Bradford KJ., Hilhorst HWM., Nonogaki H. Seeds: Physiology of Development, Germination and Dormancy. 3rd ed. New York: Springer; 2013. https://doi:10.1007/978-1-4614-4693-4

16. Chatelain E, Hundertmark M, Leprince O, Gall S Le, Satour P, Deligny-Penninck S, et al. Temporal profiling of the heat-stable proteome during late maturation of *Medicago truncatula* seeds identifies a restricted subset of late embryogenesis abundant proteins associated with longevity. Plant, Cell Environ. 2012; 35: 1440–1455. https://doi:10.1111/j.1365-3040.2012.02501.x

17. Dekkers BJW, He H, Hanson J, Willems LAJ, Jamar DCL, Cueff G, et al. The Arabidopsis Delay of Germination 1 gene affects Abscisic Acid Insensitive 5 (ABI5) expression and genetically interacts with ABI3 during Arabidopsis seed development. Plant J. 2016; 85: 451–465. https://doi:10.1111/tpj.13118

18. Hell AF, Kretzschmar FS, Simões K, Heyer AG, Barbedo CJ, Braga MR, et al. Metabolic Changes on the Acquisition of Desiccation Tolerance in Seeds of the Brazilian Native Tree Erythrina speciosa. Front Plant Sci. 2019; 10: 1–15. https://doi:10.3389/fpls.2019.01356

19. Hoekstra FA, Golovina EA, Buitink J. Mechanism of plant desiccation tolerance. Trends Plant

Sci. 2001; 6: 431–438. https://doi:10.1016/S1360-1385(01)02052-0

20. Oliver MJ, Farrant JM, Hilhorst HWM, Mundree S, Williams B, Bewley JD. Desiccation Tolerance: Avoiding Cellular Damage during Drying and Rehydration. Annu Rev Plant Biol. 2020; 71: 435–460. https://doi:10.1146/annurev-arplant-071219-105542

21. Sano N, Rajjou L, North HM, Debeaujon I, Marion-Poll A, Seo M. Staying alive: Molecular aspects of seed longevity. Plant Cell Physiol. 2016; 57: 660–674. https://doi:10.1093/pcp/pcv186

22. Moreno L de A, Fonseca de Oliveira GR, Batista TB, Bossolani JW, Ducatti KR, Guimarães CC, et al. Quality of cowpea seeds: A food security strategy in the tropical environment. PLoS One. 2022; 17: 1-13. https://doi:10.1371/journal.pone.0276136

23. Finch-Savage WE, Bassel GW. Seed vigour and crop establishment: Extending performance beyond adaptation. J Exp Bot. 2016; 67: 567–591. https://doi:10.1093/jxb/erv490

24. Fonseca de Oliveira GR, Amaral da Silva EA. Tropical peanut maturation scale for harvesting seeds with superior quality. Front Plant Sci. 2024; 15: 1–15. https://doi:10.3389/fpls.2024.1376370

25. Routaboul JM, Kerhoas L, Debeaujon I, Pourcel L, Caboche M, Einhorn J, et al. Flavonoid diversity and biosynthesis in seed of *Arabidopsis thaliana*. Planta. 2006; 224: 96–107. https://doi:10.1007/s00425-005-0197-5

26. Wan L, Lei Y, Yan L, Liu Y, Pandey MK, Wan X, et al. Transcriptome and metabolome reveal redirection of flavonoids in a white testa peanut mutant. BMC Plant Biol. 2020; 20: 1–19. https://doi:10.1186/s12870-020-02383-7

27. USDA. World agricultural production. Peanut Area, Yield and Production. Dec 2025: 39–42. Access date: 03 January 2025. https://apps.fas.usda.gov/psdonline/circulars/production.pdf

28. Stalker HT, Wilson RF. Peanuts: Genetics, Processing, and Utilization. Academic Press and AOCS Press. First Edition. 2016. 498 pages.

29. Saito K, Yonekura-Sakakibara K, Nakabayashi R, Higashi Y, Yamazaki M, Tohge T, et al.

The flavonoid biosynthetic pathway in Arabidopsis: Structural and genetic diversity. Plant Physiol Biochem. 2013; 72: 21–34. https://doi:10.1016/j.plaphy.2013.02.001

30. Ambawat S, Sharma P, Yadav NR, Yadav RC. MYB transcription factor genes as regulators for plant responses: An overview. Physiol Mol Biol Plants. 2013; 19: 307–321. https://doi:10.1007/s12298-013-0179-1

31. Biswas D, Gain H, Mandal A. MYB transcription factor: A new weapon for biotic stress tolerance in plants. Plant Stress. 2023; 10: 1-9. https://doi:10.1016/j.stress.2023.100252

32. Mehrtens F, Kranz H, Bednarek P, Weisshaar B. The Arabidopsis transcription factor MYB12 is a flavonol-specific regulator of phenylpropanoid biosynthesis. Plant Physiol. 2005; 138: 1083–1096. doi:10.1104/pp.104.058032

33. Wang N, Xu H, Jiang S, Zhang Z, Lu N, Qiu H, et al. MYB12 and MYB22 play essential roles in proanthocyanidin and flavonol synthesis in red-fleshed apple (*Malus sieversii f. niedzwetzkyana*). Plant J. 2017; 90: 276–292. https://doi:10.1111/tpj.13487

34. Dhakarey R, Yaritz U, Tian L, Amir R. A Myb transcription factor, PgMyb308-like, enhances the level of shikimate, aromatic amino acids, and lignins, but represses the synthesis of flavonoids and hydrolyzable tannins, in pomegranate (*Punica granatum* L.). Hortic Res. 2022; 9: 1–13. https://doi:10.1093/hr/uhac008

35. Choi SJ, Lee Z, Kim S, Jeong E, Shim JS. Modulation of lignin biosynthesis for drought tolerance in plants. Front Plant Sci. 2023; 14: 1–14. https://doi:10.3389/fpls.2023.1116426

36. LaFountain AM, Yuan Y. Repressors of anthocyanin biosynthesis. New Phytol. 2021; 231: 933–949. https://doi:10.1111/nph.17397

37. Verdier J, Lalanne D, Pelletier S, Torres-Jerez I, Righetti K, Bandyopadhyay K, et al. A regulatory network-based approach dissects late maturation processes related to the acquisition of desiccation tolerance and longevity of *Medicago truncatula* seeds. Plant Physiol. 2013; 163: 757–774. https://doi:10.1104/pp.113.222380

38. Berka M, Kopecká R, Berková V, Brzobohatý B, Černý M. Regulation of heat shock proteins

70 and their role in plant immunity. J Exp Bot. 2022; 73: 1894–1909. https://doi:10.1093/jxb/erab549

39. Charng YY, Liu HC, Liu NY, Chi WT, Wang CN, Chang SH, et al. A heat-inducible transcription factor, HsfA2, is required for extension of acquired thermotolerance in Arabidopsis. Plant Physiol. 2007; 143: 251–262. https://doi:10.1104/pp.106.091322

40. Zinsmeister J, Lalanne D, Terrasson E, Chatelain E, Vandecasteele C, Ly Vu B, et al. ABI5 is a regulator of seed maturation and longevity in legumes. Plant Cell. 2016; 28: 2735–2754. https://doi:10.1105/tpc.16.00470

41. Bizouerne E, Buitink J, Vu BL, Vu JL, Esteban E, Pasha A, et al. Gene co-expression analysis of tomato seed maturation reveals tissue-specific regulatory networks and hubs associated with the acquisition of desiccation tolerance and seed vigour. BMC Plant Biol. 2021; 21: 1–23. https://doi:10.1186/s12870-021-02889-8

42. ISTA. International Rules for Seed Analysis. International rules for seed testing. 2020. edn Vol. 50 (Bassersdorf, Suíça: Zürischstr), CH–8303.

43. Fonseca de Oliveira GR, Hirai WY, Ferreira DS, Silva KPOM da, Silva GC, Moraes TB, et al. Spectroscopy Technologies to Screen Peanut Seeds with Superior Vigor Through "Chemical Fingerprinting." Agronomy. 2024; 14: 1-17. https://doi:10.3390/agronomy14112529

44. Joosen RVL, Kodde J, Willems LAJ, Ligterink W, Van Der Plas LHW, Hilhorst HWM. Germinator: A software package for high-throughput scoring and curve fitting of Arabidopsis seed germination. Plant J. 2010; 62: 148–159. https://doi:10.1111/j.1365-313X.2009.04116.x

45. Rossetto CAV, Lima T de M, Guimarães E da C. Envelhecimento acelerado e deterioração controlada em sementes de amendoim. Pesqui Agropecuária Bras. 2004; 39: 795–801. https://doi:10.1590/s0100-204x2004000800010

46. Krzyzanowski FC, França-Neto JB, Gomes-Junior, F. G., Nakagawa J. Testes de vigor baseado em desempenho de plântulas. In: Krzyzanowski, F C, Vieira, R D, França-Neto, 549 J B, Marcos-Filho, J Vigor de sementes: conceitos e testes. 2ed. Londrina, Paraná, Brazil; 2020.

pp. 79–140.
47. Nakagawa J. Testes de vigor baseados no crescimento de plântulas. In: Vieira, R.D.; Carvalho, N.M. Testes de vigor em sementes. Jaboticabal: FUNEP; 1994.
48. Oliveira NM, Medeiros AD de, Nogueira M de L, Arthur V, Mastrangelo T de A, Barboza da Silva C. Hormetic effects of low-dose gamma rays in soybean seeds and seedlings: A detection technique using optical sensors. Comput Electron Agric. 2021; 187: 1-9. https://doi:10.1016/j.compag.2021.106251
49. Batista TB, Fernandez GJ, Da Silva TA, Maia J, Da Silva EAA. Transcriptome analysis in osmo-primed tomato seeds with enhanced longevity by heat shock treatment. AoB Plants. 2020; 12: 1–10. https://doi:10.1093/aobpla/plaa041
50. Chen S, Zhou Y, Chen Y, Gu J. Fastp: An ultra-fast all-in-one FASTQ preprocessor. Bioinformatics. 2018; 34: i884–i890. https://doi:10.1093/bioinformatics/bty560
51. Dobin A, Davis CA, Schlesinger F, Drenkow J, Zaleski C, Jha S, et al. STAR: Ultrafast universal RNA-seq aligner. Bioinformatics. 2013; 29: 15–21. https://doi:10.1093/bioinformatics/bts635
52. Bertioli DJ, Jenkins J, Clevenger J, Dudchenko O, Gao D, Seijo G, et al. The genome sequence of segmental allotetraploid peanut Arachis hypogaea. Nat Genet. 2019; 51: 877–884. https://doi:10.1038/s41588-019-0405-z
53. Goodstein DM, Shu S, Howson R, Neupane R, Hayes RD, Fazo J, et al. Phytozome: A comparative platform for green plant genomics. Nucleic Acids Res. 2012; 40: 1178–1186. https://doi:10.1093/nar/gkr944
54. Love MI, Huber W, Anders S. Moderated estimation of fold change and dispersion for RNA-seq data with DESeq2. Genome Biol. 2014; 15: 1–21. https://doi:10.1186/s13059-014-0550-8
55. Tian T, Liu Y, Yan H, You Q, Yi X, Du Z, et al. AgriGO v2.0: A GO analysis toolkit for the agricultural community. Nucleic Acids Res. 2017; 45: 122–129. http://doi:10.1093/nar/gkx382
56. Untergasser A, Cutcutache I, Koressaar T, Ye J, Faircloth BC, Remm M, et al. Primer3-new

capabilities and interfaces. Nucleic Acids Res. 2012; 40: 1–12. http://doi:10.1093/nar/gks596

57. Livak KJ, Schmittgen TD. Analysis of relative gene expression data using real-time quantitative PCR and the 2-ΔΔCT method. Methods. 2001; 25: 402–408. http://doi:10.1006/meth.2001.1262

58. Brand Y, Hovav R. Identification of Suitable Internal Control Genes for Quantitative Real-Time PCR Expression Analyses in Peanut (*Arachis hypogaea*) . Peanut Sci. 2010; 37: 12–19. http://doi:10.3146/ps09-014.1

59. Morgante CV, Guimarães PM, Martins ACQ, Araújo ACG, Leal-Bertioli SCM, Bertioli DJ, et al. Reference genes for quantitative reverse transcription-polymerase chain reaction expression studies in wild and cultivated peanut. BMC Res Notes. 2011; 4: 7–12. http://doi:10.1186/1756-0500-4-339

60. Anderson MJ. Permutational Multivariate Analysis of Variance (PERMANOVA). Wiley StatsRef: Statistics Reference Online. Wiley; 2017. pp. 1–15. http://doi:10.1002/9781118445112.stat07841

61. R Core Team. A Language and Environment for Statistical Computing: R Foundation for Statistical Computing. Vienna, Austria. 2024.

62. Jiménez N, Bohuon P, Dornier M, Bonazzi C, Pérez AM, Vaillant F. Effect of water activity on anthocyanin degradation and browning kinetics at high temperatures (100-140ºC). Food Res Int. 2012; 47: 106–115. http://doi:10.1016/j.foodres.2012.02.004

63. Mattioli R, Francioso A, Mosca L, Silva P. Anthocyanins: A Comprehensive Review of Their Chemical Properties and Health Effects on Cardiovascular and Neurodegenerative Diseases. Molecules. 2020; 25. http://doi:10.3390/molecules25173809

64. Berardino JD., Marmagne A., Berger A., Yoshimoto K., Cueff G., Chardon F., et al. Autophagy controls resource allocation and protein storage accumulation in Arabidopsis seeds. J Exp Bot. 2018; 69: 1403–1414. http://doi:10.1093/jxb/ery012

65. Setter TL, Flannigan BA. Water deficit inhibits cell division and expression of transcripts

involved in cell proliferation and endoreduplication in maize endosperm. J Exp Bot. 2001; 52: 1401–1408. http://doi:10.1093/jexbot/52.360.1401

66. Aldana AB, Fites RC, Pattee HE. Changes in nucleic acids, protein and ribonuclease activity during maturation of peanut seeds. Plant Cell Physiol. 1972; 13: 515–521. http://doi:10.1093/oxfordjournals.pcp.a074764

67. Wang J, Li D, Peng Y, Cai M, Liang Z, Yuan Z, et al. The Anthocyanin Accumulation Related ZmBZ1, Facilitates Seedling Salinity Stress Tolerance via ROS Scavenging. Int J Mol Sci. 2022; 23. http://doi:10.3390/ijms232416123

68. Cerqueira JVA, de Andrade MT, Rafael DD, Zhu F, Martins SVC, Nunes-Nesi A, et al. Anthocyanins and reactive oxygen species: a team of rivals regulating plant development? Plant Mol Biol. 2023; 112: 213–223. http://doi:10.1007/s11103-023-01362-4

69. Pattee HE, Johns EB, Singleton JA, Sanders TH. Composition Changes of Peanut Fruit Parts During Maturation. Peanut Sci. 1974; 1: 57–62. http://doi:10.3146/i0095-3679-1-2-6

70. Liu Y, Tikunov Y, Schouten RE, Marcelis LFM, Visser RGF, Bovy A. Anthocyanin biosynthesis and degradation mechanisms in Solanaceous vegetables: A review. Front Chem. 2018; 6. http://doi:10.3389/fchem.2018.00052

71. Wehmeyer N, Vierling E. The Expression of Small Heat Shock Proteins in Seeds Responds to Discrete Developmental Signals and Suggests a General Protective Role in Desiccation Tolerance. Plant Physiol. 2000; 122: 1099–1108. https://doi.org/10.1104/pp.122.4.1099

72. Artur MAS, Zhao T, Ligterink W, Schranz E, Hilhorst HWM. Dissecting the genomic diversification of late embryogenesis abundant (LEA) protein gene families in plants. Genome Biol Evol. 2019; 11: 459–471. https://doi:10.1093/gbe/evy248

73. Zhang C, Yao X, Ren H, Chang J, Wang K. RNA-Seq Reveals Flavonoid Biosynthesis-Related Genes in Pecan (*Carya illinoinensis*) Kernels. J Agric Food Chem. 2019; 67: 148–158. https://doi:10.1021/acs.jafc.8b05239

74. Xu Y, Zou J, Zheng H, Xu M, Zong X, Wang L. RNA-seq transcriptome analysis of rice

primary roots reveals the role of flavonoids in regulating the rice primary root growth. Genes (Basel). 2019; 10: 1-16. https://doi:10.3390/genes10030213

75. Erb M, Kliebenstein DJ. Plant Secondary Metabolites as Defenses, Regulators, and Primary Metabolites: The Blurred Functional Trichotomy. Plant Physiol. 2020; 184: 39–52. https://doi:10.1104/PP.20.00433

76. Dao TTH, Linthorst HJM, Verpoorte R. Chalcone synthase and its functions in plant resistance. Phytochem Rev. 2011; 10: 397–412. https://doi:10.1007/s11101-011-9211-7

77. Deng X, Bashandy H, Ainasoja M, Kontturi J, Pietiäinen M, Laitinen RAE, et al. Functional diversification of duplicated chalcone synthase genes in anthocyanin biosynthesis of Gerbera hybrida. New Phytol. 2014; 201: 1469–1483. https://doi:10.1111/nph.12610

78. Ma W, Qayyum Z, Noureen F, Khan M, Amir R, Khan M, et al. Identification and Expression Analysis of Stilbene Synthase Genes in Arachis hypogaea in Response to Methyl Jasmonate and Salicylic Acid Induction. Plants. 2022; 11. https://doi:10.3390/plants11131776

79. Xia H, Zhu L, Zhao C, Li K, Shang C, Hou L, et al. Comparative transcriptome analysis of anthocyanin synthesis in black and pink peanut. Plant Signal Behav. 2020; 15. https://doi:10.1080/15592324.2020.1721044

80. Zhang W, Zhang B wen, Deng J fu, Li L, Yi T yong, Hong Y yun. The resistance of peanut to soil-borne pathogens improved by rhizosphere probiotics under calcium treatment. BMC Microbiol. 2021; 21: 1–13. https://doi:10.1186/s12866-021-02355-3

81. Yao Y, Gao S, Ding X, Zhang Q, Li P. Topography effect on Aspergillus flavus occurrence and aflatoxin B1 contamination associated with peanut. Curr Res Microb Sci. 2021; 2: 1-10. https://doi:10.1016/j.crmicr.2021.100021

82. Liu K, Liu Y, Chen F. Effect of storage temperature on lipid oxidation and changes in nutrient contents in peanuts. Food Sci Nutr. 2019; 7: 2280–2290. https://doi:10.1002/fsn3.1069

83. López PL, Marchesino MA, Grosso NR, Olmedo RH. Comparative study of accelerated assays for determination of equivalent days in the shelf life of roasted high oleic peanuts: Chemical

and volatile oxidation indicators in accelerated and room temperature conditions. Food Chem. 2022; 373. https://doi:10.1016/j.foodchem.2021.131479

84. Oenel A, Fekete A, Krischke M, Faul SC, Gresser G, Havaux M, et al. Enzymatic and non-enzymatic mechanisms contribute to lipid oxidation during seed aging. Plant Cell Physiol. 2017; 58: 925–933. https://doi:10.1093/pcp/pcx036

85. Mantegazza O, Gregis V, Mendes MA, Morandini P, Alves-Ferreira M, Patreze CM, et al. Analysis of the arabidopsis REM gene family predicts functions during flower development. Ann Bot. 2014; 114: 1507–1515. https://doi:10.1093/aob/mcu124

86. Liu H, Li J, Xu C, Liu H, Zhao Z. Characterization and expression analysis of the B3 gene family during seed development in Akebia trifoliata. BMC Genomics. 2024; 25: 1–20. https://doi:10.1186/s12864-024-10981-0

87. Delahaie J, Hundertmark M, Bove J, Leprince O, Rogniaux H, Buitink J. LEA polypeptide profiling of recalcitrant and orthodox legume seeds reveals ABI3-regulated LEA protein abundance linked to desiccation tolerance. J Exp Bot. 2013; 64: 4559–4573. https://doi:10.1093/jxb/ert274

88. Wei C, Jiao Q, Agathokleous E, Liu H, Li G, Zhang J, et al. Hormetic effects of zinc on growth and antioxidant defense system of wheat plants. Sci Total Environ. 2022; 807: 1-13. https://doi:10.1016/j.scitotenv.2021.150992

89. Waters ER. The evolution, function, structure, and expression of the plant sHSPs. J Exp Bot. 2013; 64: 391–403. https://doi:10.1093/jxb/ers355

90. Brasileiro ACM, Gimenes MA, Pereira BM, Mota APZ, Aguiar MN, Martins ACQ, et al. The Stilbene Synthase Family in Arachis: A Genome-Wide Study and Functional Characterization in Response to Stress. Genes (Basel). 2023; 14:1-24. https://doi:10.3390/genes14122181

91. Ji J, Yang L, Fang Z, Zhang Y, Zhuang M, Lv H, et al. Plant SWEET Family of Sugar Transporters: Structure, Evolution and Biological Functions. Biomolecules. 2022; 12:1-19 https://doi:10.3390/biom12020205

92. Bagnato L, Tosato E, Gurrieri L, Trost P, Forlani G, Sparla F. Arabidopsis thaliana Sucrose Phosphate Synthase A2 Affects Carbon Partitioning and Drought Response. Biology (Basel). 2023; 12:1-16. https://doi:10.3390/biology12050685

93. Zinsmeister J, Leprince O, Buitink J. Molecular and environmental factors regulating seed longevity. Biochem J. 2020; 477: 305–323. https://doi:10.1042/BCJ20190165

94. Okada MH, Oliveira GRF, Sartori MMP, Crusciol CAC, Nakagawa J, Amaral da Silva EA. Acquisition of the physiological quality of peanut (*Arachis hypogaea* L.) seeds during maturation under the influence of the maternal environment. PLoS One. 2021; 16:1-15. https://doi:10.1371/journal.pone.0250293

95. Bonku R, Yu J. Health aspects of peanuts as an outcome of its chemical composition. Food Sci Hum Wellness. 2020; 9: 21–30. https://doi:10.1016/j.fshw.2019.12.005

96. Neelakandan AK, Subedi B, Traore SM, Binagwa P, Wright DA, He G. Base Editing in Peanut Using CRISPR/nCas9. Front Genome. 2022; 4: 1–8. https://doi:10.3389/fgeed.2022.901444

97. Reed RC, Bradford KJ, Khanday I. Seed germination and vigor: ensuring crop sustainability in a changing climate. Heredity. 2022; 128: 450–459. https://doi:10.1038/s41437-022-00497-2

98. Li Y, Baldauf S, Lim EK, Bowles DJ. Phylogenetic Analysis of the UDP-glycosyltransferase Multigene Family of *Arabidopsis thaliana*. J Biol Chem. 2001; 276: 4338–4343. https://doi:10.1074/jbc.M007447200

99. Nagatoshi M, Terasaka K, Nagatsu A, Mizukami H. Iridoid-specific glucosyltransferase from Gardenia jasminoides. J Biol Chem. 2011; 286: 32866–32874. https://doi:10.1074/jbc.M111.242586

100. Cerdá-Bernad D, Valero-Cases E, Pastor JJ, Frutos MJ. Saffron bioactives crocin, crocetin and safranal: effect on oxidative stress and mechanisms of action. Crit Rev Food Sci Nutr. 2022; 62: 3232–3249. https://doi:10.1080/10408398.2020.1864279

101. Vassão DG, Wielsch N, Gomes AM de MM, Gebauer-Jung S, Hupfer Y, Svatoš A, et al. Plant

defensive β-Glucosidases resist digestion and sustain activity in the gut of a lepidopteran herbivore. Front Plant Sci. 2018; 9: 1–13. https://doi:10.3389/fpls.2018.01389

102. Morant AV, Jørgensen K, Jørgensen C, Paquette SM, Sánchez-Pérez R, Møller BL, et al. β-Glucosidases as detonators of plant chemical defense. Phytochemistry. 2008; 69: 1795–1813. https://doi:10.1016/j.phytochem.2008.03.006

103. Raychaudhuri A, Tipton PA. Cloning and expression of the gene for soybean hydroxyisourate hydrolase. Localization and implications for function and mechanism. Plant Physiol. 2002; 130: 2061–2068. https://doi:10.1104/pp.011049

104. Song JT, Koo YJ, Seo HS, Kim MC, Choi Y Do, Kim JH. Overexpression of AtSGT1, an Arabidopsis salicylic acid glucosyltransferase, leads to increased susceptibility to Pseudomonas syringae. Phytochemistry. 2008; 69: 1128–1134. https://doi:10.1016/j.phytochem.2007.12.010

105. Takenaka Y, Kato K, Ogawa-Ohnishi M, Tsuruhama K, Kajiura H, Yagyu K, et al. Pectin RG-I rhamnosyltransferases represent a novel plant-specific glycosyltransferase family. Nat Plants. 2018; 4: 669–676. https://doi:10.1038/s41477-018-0217-7

106. Jørgensen K, Morant AV, Morant M, Jensen NB, Olsen CE, Kannangara R, et al. Biosynthesis of the cyanogenic glucosides linamarin and lotaustralin in cassava: Isolation, biochemical characterization, and expression pattern of cyp71e7, the oxime-metabolizing cytochrome P450 enzyme. Plant Physiol. 2011; 155: 282–292. https://doi:10.1104/pp.110.164053

107. Ren C, Guo Y, Xie L, Zhao Z, Xing M, Cao Y, et al. Identification of UDP-rhamnosyltransferases and UDP-galactosyltransferase involved in flavonol glycosylation in Morella rubra. Hortic Res. 2022; 9: 1–14. https://doi:10.1093/hr/uhac138

108. Vannozzi A, Wong DCJ, Höll J, Hmmam I, Matus JT, Bogs J, et al. Combinatorial Regulation of Stilbene Synthase Genes by WRKY and MYB Transcription Factors in Grapevine (*Vitis vinifera* L.). Plant Cell Physiol. 2018; 59: 1043–1059. https://doi:10.1093/pcp/pcy045

109. Cui B, Pan Q, Clarke D, Villarreal MO, Umbreen S, Yuan B, et al. S-nitrosylation of the zinc

finger protein SRG1 regulates plant immunity. Nat Commun. 2018; 9: 1–12. https://doi:10.1038/s41467-018-06578-3

110. Cao Y, Li G, Wang X, Huang R, Luo J, Li M, et al. Overexpression of a Senescence-Related Gene CpSRG1 from Wintersweet (*Chimonanthus praecox*) Promoted Growth and Flowering, and Delayed Senescence in Transgenic Arabidopsis. Int J Mol Sci. 2022; 23: 1–15. https://doi:10.3390/ijms232213971

111. Gao J, Zhang R, Zheng L, Song L, Ji M, Li S, et al. Blue light receptor CRY1 regulates HSFA1d nuclear localization to promote plant thermotolerance. Cell Rep. 2023; 42: 1-19. https://doi:10.1016/j.celrep.2023.113117

112. Barrero JM, Downie AB, Xu Q, Gubler F. A role for barley CRYPTOCHROME1 in light regulation of grain dormancy and germination. Plant Cell. 2014; 26: 1094–1104. https://doi:10.1105/tpc.113.121830

113. Shao H, Dixon RA, Wang X. Crystal Structure of Vestitone Reductase from Alfalfa (*Medicago sativa* L.). J Mol Biol. 2007; 369: 265–276. https://doi:10.1016/j.jmb.2007.03.040

114. Gharabli H, Della Gala V, Welner DH. The function of UDP-glycosyltransferases in plants and their possible use in crop protection. Biotechnol Adv. 2023; 67: 1-14. https://doi:10.1016/j.biotechadv.2023.108182

115. Roscoe TJ, Vaissayre V, Paszkiewicz G, Clavijo F, Kelemen Z, Michaud C, et al. Regulation of FUSCA3 Expression During Seed Development in Arabidopsis. Plant Cell Physiol. 2019; 60: 476–487. https://doi:10.1093/pcp/pcy224

116. Yang Y, Kong Q, Lim ARQ, Lu S, Zhao H, Guo L, et al. Transcriptional regulation of oil biosynthesis in seed plants: Current understanding, applications, and perspectives. Plant Commun. 2022; 3: 1-19. https://doi:10.1016/j.xplc.2022.100328

117. Huang S, Raman AS, Ream JE, Fujiwara H, Eric Cerny R, Brown SM. Overexpression of 20-oxidase confers a gibberellin-overproduction phenotype in arabidopsis. Plant Physiol. 1998; 118: 773–781. https://doi:10.1104/pp.118.3.773

118. Plackett ARG, Powers SJ, Fernandez-Garcia N, Urbanova T, Takebayashi Y, Seo M, et al. Analysis of the developmental roles of the arabidopsis gibberellin 20-oxidases demonstrates that GA20ox1, -2, and -3 are the dominant paralogs. Plant Cell. 2012; 24: 941–960. https://doi:10.1105/tpc.111.095109

119. Seidel C, Walz A, Park S, Cohen JD, Ludwig-Müller J. Indole-3-acetic acid protein conjugates: Novel players in auxin homeostasis. Plant Biol. 2006; 8: 340–345. https://doi:10.1055/s-2006-923802

120. Walz A, Park S, Slovin JP, Ludwig-Müller J, Momonoki YS, Cohen JD. A gene encoding a protein modified by the phytohormone indoleacetic acid. Proc Natl Acad Sci USA. 2002; 99: 1718–1723. https://doi:10.1073/pnas.032450399

121. Candat A, Paszkiewicz G, Neveu M, Gautier R, Logan DC, Avelange-Macherel MH, et al. The ubiquitous distribution of late embryogenesis abundant proteins across cell compartments in Arabidopsis offers tailored protection against abiotic stress. Plant Cell. 2014; 26: 3148–3166. https://doi:10.1105/tpc.114.127316

122. Li T, Zhang Y, Wang D, Liu Y, Dirk LMA, Goodman J, et al. Regulation of Seed Vigor by Manipulation of Raffinose Family Oligosaccharides in Maize and Arabidopsis thaliana. Mol Plant. 2017; 10: 1540–1555. https://doi:10.1016/j.molp.2017.10.014

123. Sengupta S, Mukherjee S, Basak P, Majumder AL. Significance of galactinol and raffinose family oligosaccharide synthesis in plants. Front Plant Sci. 2015; 6: 1–11. https://doi:10.3389/fpls.2015.00656

124. Zhan C, Li Y, Li H, Wang M, Gong S, Ma D, et al. Phylogenomic analysis of phenylalanine ammonia-lyase (PAL) multigene family and their differential expression analysis in wheat (*Triticum aestivum* L.) suggested their roles during different stress responses. Front Plant Sci. 2022; 13: 1–15. https://doi:10.3389/fpls.2022.982457

125. Kim DS, Hwang BK. An important role of the pepper phenylalanine ammonia-lyase gene (PAL1) in salicylic acid-dependent signalling of the defence response to microbial pathogens.

J Exp Bot. 2014; 65: 2295–2306. https://doi:10.1093/jxb/eru109

126. Cairns NG, Pasternak M, Wachter A, Cobbett CS, Meyer AJ. Maturation of Arabidopsis Seeds Is Dependent on Glutathione Biosynthesis within the Embryo. Plant Physiol. 2006; 141: 446–455. https://doi:10.1104/pp.106.077982.446

127. Niñoles R, Planes D, Arjona P, Ruiz-Pastor C, Chazarra R, Renard J, et al. Comparative analysis of wild-type accessions reveals novel determinants of Arabidopsis seed longevity. Plant Cell Environ. 2022; 45: 2708–2728. https://doi:10.1111/pce.14374

Fig 1. Seed physiology during peanut development stages (cultivar IAC OL3). All means have standard deviations. Different letters on the bars are significantly different from each other by Scott Knott test (p-value ≤ 0.05). Asterisks (**) in all graphs signify the result of 1% significance by F-test (ANOVA). The dots along the bars are the biological replicates for each seed test (A-N).

Fig 2. Principal component analysis (PCA) performed with all seed physiology data and anthocyanin dynamics (A). Anthocyanin index, obtained by multispectral imaging (B). Spearman correlation between anthocyanin and seed quality components (C).

Fig 3. RNAseq analysis focused on flavonoid biosynthesis. PCA of transcriptome data and plotted to track significantly expressed genes from early and late stages (A-B). Gene ontology (GO) analysis shows the groups of differently expressed genes (DEGs) in each enriched GO analysis, biological process (C). Heatmap of genes associated with anthocyanin synthesis and the list of key genes chosen for RT-qPCR study (C-D). Gene expression data in the heat map was transformed using the DESeq2 regularized log (rlog) method, which stabilizes variance across mean expression levels.

Fig 4. Flavonoid biosynthesis pathway and the key genes along it. Relative gene expression by RT-qPCR during seed development stages (A-G). All means have standard deviations. Different letters

on the bars are significantly different from each other by Scott Knott test (p-value ≤ 0.05). Asterisks (**) in all graphs signify the result of 1% significance by F-test (ANOVA). The dots along the bars are the biological replicates for each seed test. Average Gini decrease performed to highlight the most relevant gene for predicting peanut seed development, *AhCHS* (H).

Fig 5. Peanut seed development overview: physiological and molecular approach (A and B). Gene expression was obtained by gene counts normalized (DESeq2 size factor method), which adjusts for differences in sequencing depth between samples. The peanut fruit and seed images are original (cultivar IAC OL3).

Table 1. The toolkit for resilience in peanut seed during late maturation. Downregulated genes associated with secondary metabolism found in the RNAseq (Flavonoid biosynthesis, GO: 0009813).

**Gene ID** [1]	**Descriptions** [2]	**Alias**	**Log_2 Fold Change** [3]	**Biological function**	**Literature**
R5P48D	UDP-glycosyltransferase 83A1	*UGT83A1*	-8.7478	Defense responses	[98]
GBEE9Q	UDP-glucose iridoid glucosyltransferase	*UGT85A24*	-6.4866	Resilience responses, iridoids	[99]
12FNT9	Crocetin glucosyltransferase	*UGT75L6*	-4.5942	Antioxidant	[100]
G9Z134	beta-glucosidase 10	*BGLU10*	-4.5652	Chemical defense, R2R3-MYB	[101]
2A3E3X	beta-glucosidase 11	*BGLU11*	-3.6176	Chemical defense, phytoanticipins	[102]
QQMN09	hydroxyisourate hydrolase	*HIUHase*	-3.1856	Ureide pathway, nitrogen	[103]
65UHA6	pathogen-inducible salicylic acid glucosyltransferase	*SGT1*	-3.1065	Pathogen control	[104]
Q8XZQ6	putative UDP-rhamnose:rhamnosyltransferase 1	*RRT1*	-3.0748	Structural cell wall, pectin	[105]
Y6LS2L	linamarin synthase 2	*UGT85K5*	-2.3463	Chemical defense, cyanogenic	[106,107]
YS6PIM	putative stilbene synthase 2	*STS2*	-2.3463	Antioxidant, resveratrol	[78,90,108]
ZH3HJS	Senescence-Related Gene 1	*SRG1*	-2.2242	Self-immunity	[109,110]
VS4P4P	cryptochrome-1	*CRY1*	-2.1990	Thermotolerance, photoreceptor	[111,112]
K47810	vestitone reductase	*VR*	-2.1856	Antimicrobial	[113]
GYU2ZH	UDP-glycosyltransferase 71K2	*UGT71K2*	-2.1312	Stress tolerance	[114]

[1] arahy.Tifrunner.gnm1.ann1.Gene ID
[2] Phytozome (https://phytozome-next.jgi.doe.gov/) and NCBI (https://blast.ncbi.nlm.nih.gov/Blast.cgi) for *A. hypogaea*
[3] Downregulated genes in the late stages (R7, R8 and R9) according to DEG construction (Early x Late).

Table 2. Key molecular players during peanut seed maturation phases from RNAseq data.

Gene ID [1]	Descriptions [2]	Alias	Log_2 Fold Change [3]	Biological functions	Literature
**Early Stages – Seed water content is about 70–65%**					
63C1KK	abscisic acid-insensitive 5-like protein 1	*ABI5*	6.8163	Master regulator of LEA, RFO, HSP	[40]
4K8YXT	B3 domain-containing transcription factor ABI3	*ABI3*	2.0705	LEA regulation, seed maturation	[87]
108MS0	B3 domain-containing protein REM23	*REM23*	6.3784	early seed development aspects	[85,86]
D9C186	B3 domain-containing transcription factor FUS3	*FUS3*	3.8649	seed development, lipid metabolism	[115,116]
0HHX3D	Delay of Germination protein-like 4	*DOG1*	2.1621	dormancy induction, seed maturation	[17]
4TCZ1X	gibberellin 20 oxidase 1	*GA20Ox1*	6.9478	gibberellin biosynthesis, seed development	[117,118]
03QWZ4	indole-3-acetic acid-induced protein	*ARG2*	2.2970	auxin homeostasis, seed development	[119,120]
EHR7LK	late embryogenesis abundant protein	*LEA3*	6.6032	protein stabilization, molecular protection	[16,72]
L9LT8B	late embryogenesis abundant protein	*LEA2*	2.1096		
DBZB80	galactinol--sucrose galactosyltransferase 5	*GSGT5*	6.84.51	raffinose, protect cell membranes	[18,19]
BM3N3L	18.1 kDa class I heat shock protein	*HSP18*	4.4017	chaperone activity, protect membranes from desiccation, thermotolerance	[38,39,89]
ECZ3V7	heat shock 70 kDa protein	*HSP70*	2.6403		
ABBA5Z	heat shock transcription factor A-2	*HSFA2*	2.5362		
QR3Y2J	small heat shock protein, chloroplastic	*HSP21*	2.0982		
**Late Stages - Seed water content is about 45–30%**					
1PU3AF	late embryogenesis abundant protein	*LEA5*	-3.1330	chaperone-like functions, proteins and membranes stabilization	[16,121]
5K3X9F	desiccation protectant protein Lea14	*LEA14*	-3.2242		
F8TXPA	Lea protein D-29	*LEA29*	-2.0009		
LY00QK	Lea protein D-34	*LEA34*	-5.0829		
V2AG0S	18.5 kDa class I heat shock protein	*HSP19*	-2.3871	chaperone-like functions, seed longevity	[10,11]
Y4YZQQ	26.5 kDa heat shock protein, mitochondrial	*HSP27*	-2.0878		
2BYT27	heat shock 70 kDa protein, mitochondrial	*HSP70*	-2.433		
1YYJ7Z	galactinol--sucrose galactosyltransferase	*GSGT*	-4.5010	raffinose biosynthesis	[122,123]
B363MR	sucrose-phosphate synthase 2	*SPS2*	-3.0305	sucrose biosynthesis, sugar transporters	[91,92]
6J07HI	bidirectional sugar transporter	*SWEET6b*	-2.2265		
S5JADZ	sugar transport protein 10	*STP10*	-4.2315		
XH4U3Y	Phenylalanine ammonia-lyase	*PAL*	-2.4316	Secondary metabolism	[124,125]
YZ4W1Q	stilbene synthase 3-like	*STS3*	-6.2299	Antioxidant, ROS scavengers	[78,90]
AS50A0	glutathione reductase, cytosolic	*GSH*	-2.0845	Antioxidant, ROS scavengers, longevity	[126,127]

[1] arahy.Tifrunner.gnm1.ann1.Gene ID.
[2] Upregulated: Early stages (R5 and R6); Downregulated: late stages (R7, R8 and R9).
[3] Phytozome (https://phytozome-next.jgi.doe.gov/) and NCBI (https://blast.ncbi.nlm.nih.gov/Blast.cgi) for *A. hypogaea*

Fig 1

Click here to access/download;Figure;Fig 1.tif

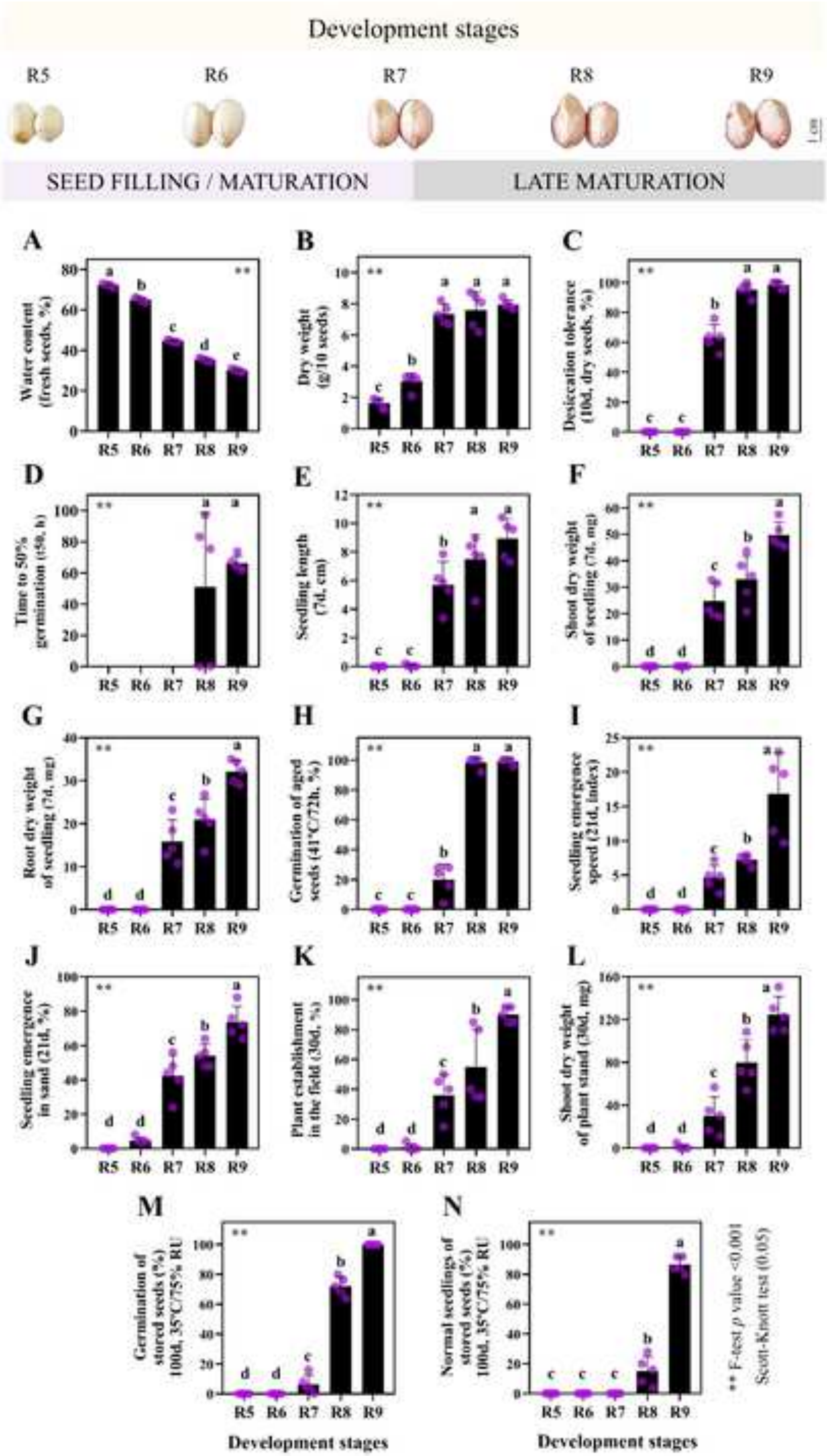

Fig 2

Click here to access/download;Figure;Fig 2.tif

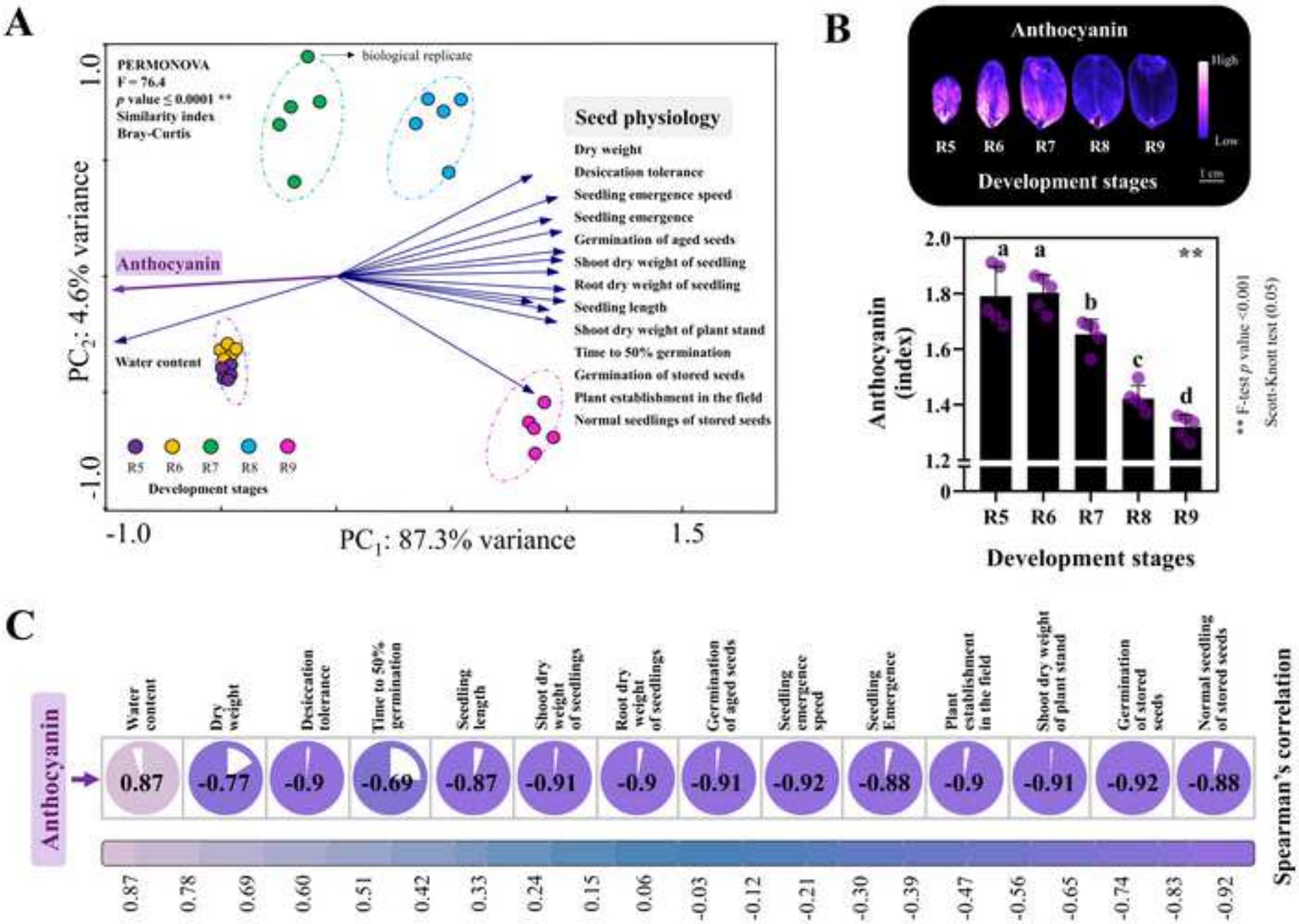

Fig 3

Click here to access/download;Figure;Fig 3.tif

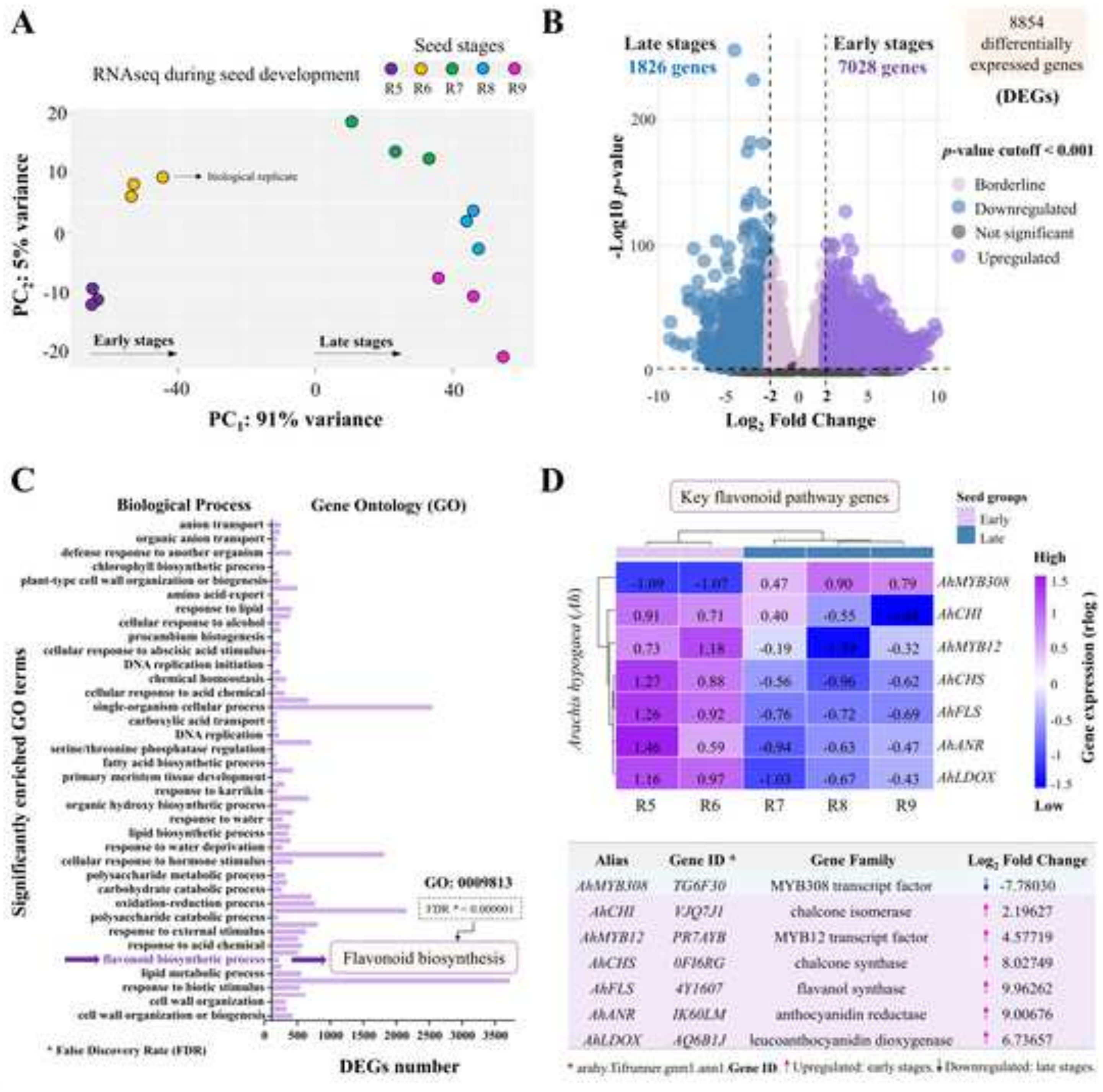

Fig 4

Click here to access/download;Figure;Fig 4.tif

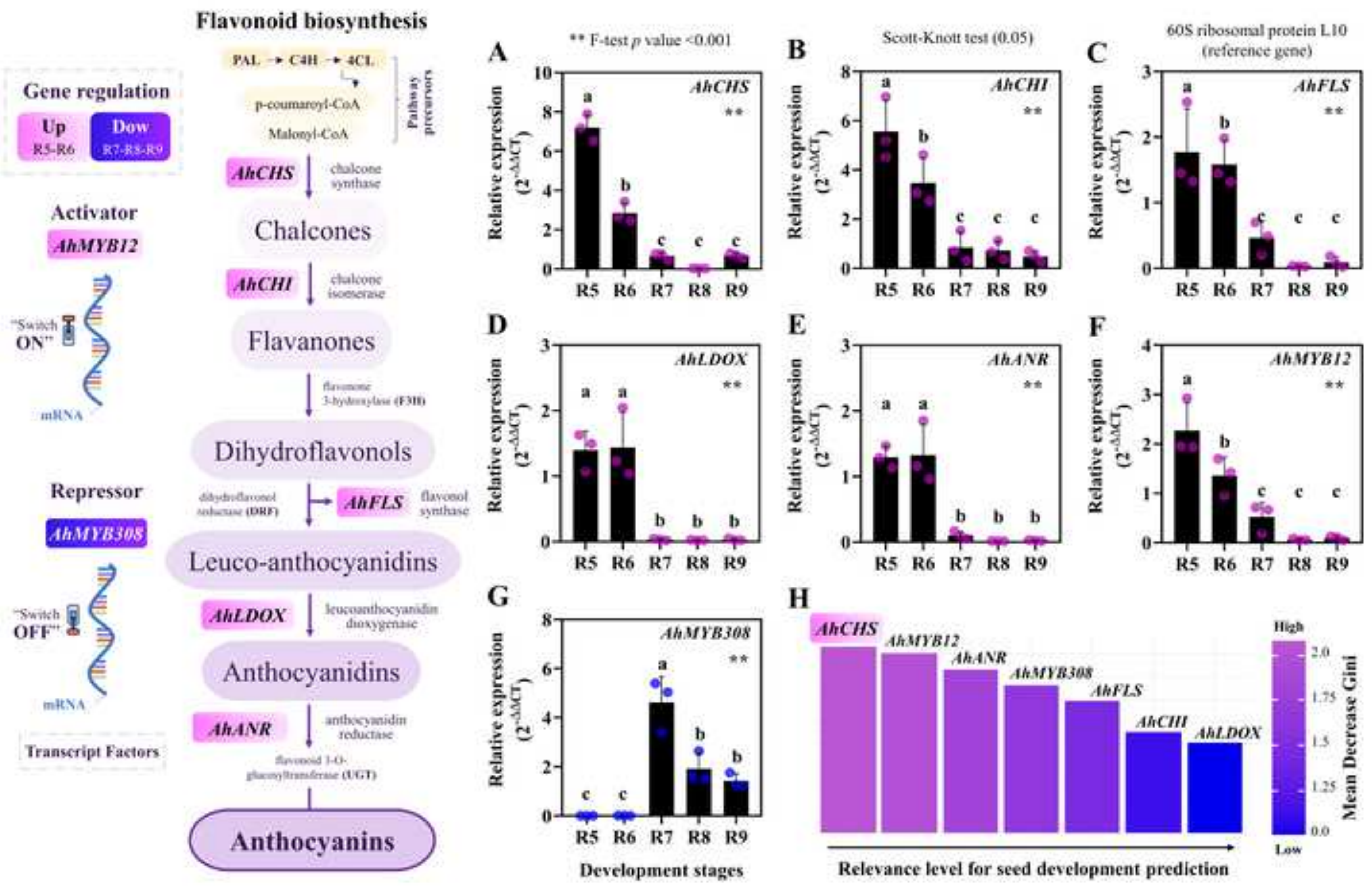

Fig 5

Click here to access/download;Figure;Fig 5.tif

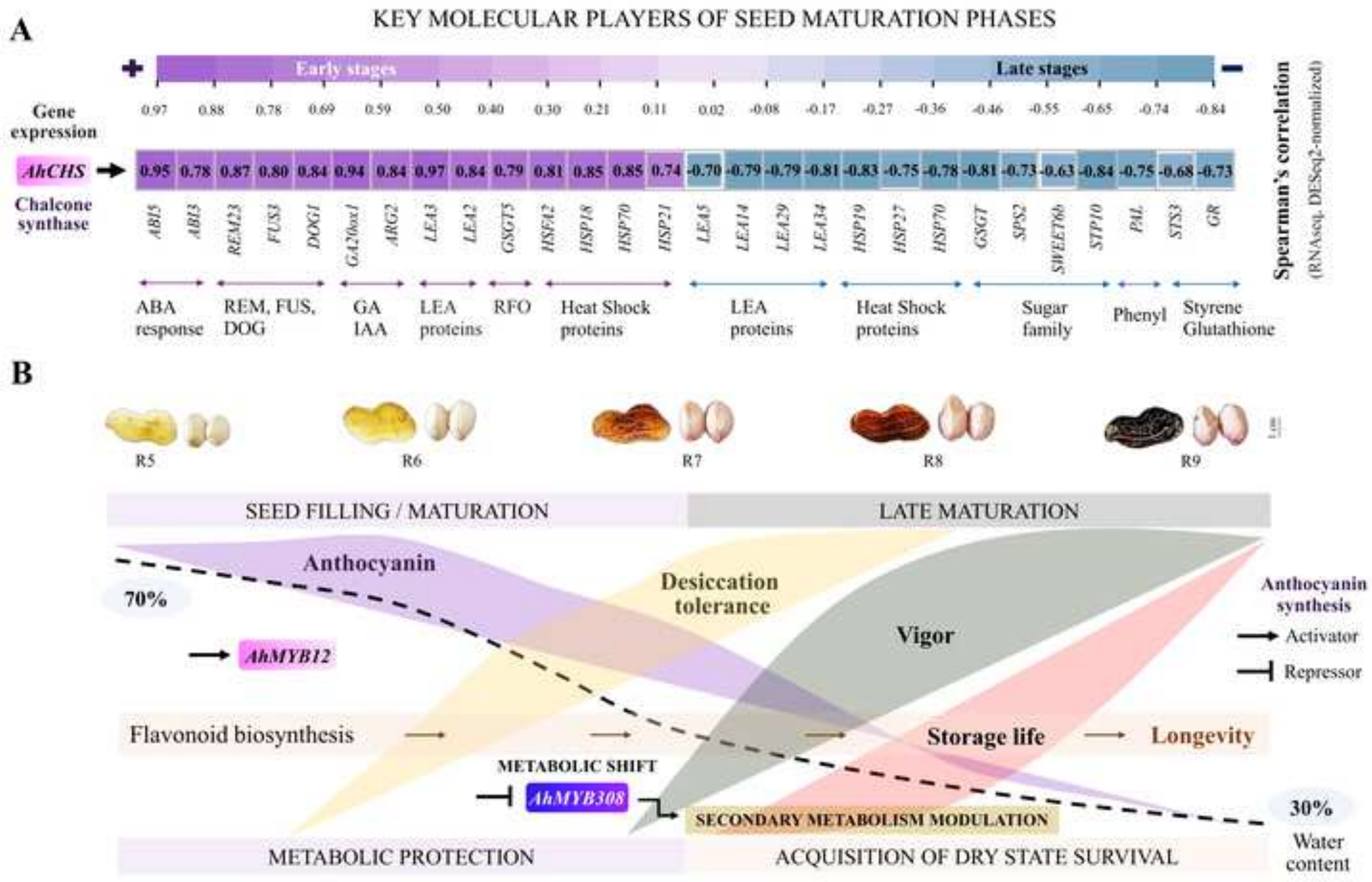

Click here to access/download
Supporting Information - Compressed/ZIP File Archive
Supporting Information.zip
